# Apoptosis-modulatory miR-361-3p as a novel treatment target in endocrine-responsive and endocrine-resistant breast cancer

**DOI:** 10.1530/JOE-22-0229

**Published:** 2023-02-11

**Authors:** J N Zamarbide Losada, E Sulpice, S Combe, G S Almeida, D A Leach, J Choo, L Protopapa, M P Hamilton, S McGuire, X Gidrol, C L Bevan, C E Fletcher

**Affiliations:** 1 Imperial Centre for Translational and Experimental Medicine, Department of Surgery & Cancer, Imperial College London, Hammersmith Hospital, London, UK; 2 Université Grenoble Alpes, CEA, INSERM, BIG, BGE, Grenoble, France; 3 Department of Molecular and Cellular Biology, Baylor College of Medicine, Houston, Texas, USA

**Keywords:** MicroRNA, breast cancer, apoptosis, drug resistance, cell cycle, non-coding RNA

## Abstract

Breast cancer (BC) is the most diagnosed cancer in women worldwide. In estrogen receptor (ER)-positive disease, anti-estrogens and aromatase inhibitors (AI) improve patient survival; however, many patients develop resistance. Dysregulation of apoptosis is a common resistance mechanism; thus, agents that can reinstate the activity of apoptotic pathways represent promising therapeutics for advanced drug-resistant disease. Emerging targets in this scenario include microRNAs (miRs). To identify miRs modulating apoptosis in drug-responsive and -resistant BC, a high-throughput miR inhibitor screen was performed, followed by high-content screening microscopy for apoptotic markers. Validation demonstrated that miR-361-3p inhibitor significantly increases early apoptosis and reduces proliferation of drug-responsive (MCF7), plus AI-/antiestrogen-resistant derivatives (LTED, TamR, FulvR), and ER- cells (MDA-MB-231). Importantly, proliferation-inhibitory effects were observed *in vivo* in a xenograft model, indicating the potential clinical application of miR-361-3p inhibition. RNA-seq of tumour xenografts identified FANCA as a direct miR-361-3p target, and validation suggested miR-361-3p inhibitor effects might be mediated in part through FANCA modulation. Moreover, miR-361-3p inhibition resulted in p53-mediated G1 cell cycle arrest through activation of p21 and reduced BC invasion. Analysis of publicly available datasets showed miR-361-3p expression is significantly higher in primary breast tumours vspaired normal tissue and is associated with decreased overall survival. In addition, miR-361-3p inhibitor treatment of BC patient explants decreased levels of miR-361-3p and proliferation marker, Ki67. Finally, miR-361-3p inhibitor showed synergistic effects on BC growth when combined with PARP inhibitor, Olaparib. Together, these studies identify miR-361-3p inhibitor as a potential new treatment for drug-responsive and -resistant advanced BC.

## Introduction

Breast cancer (BC) is the second leading cause of cancer mortality in women worldwide (1). Estrogen receptor alpha (ERα) is present in 75% of BC cases and is a dominant driver of oncogenesis in this disease subtype (Perou *et al.* 2000). ERα is a ligand-activated nuclear receptor transcription factor that, upon estrogen binding, translocates to the nucleus, where it associates with estrogen response elements in promoter and enhancer regions of target genes to activate transcriptional signatures associated with proliferation, cell cycle progression, apoptotic evasion and other tumourigenic processes. Hence, endocrine therapy – aimed either at directly competing with estrogen for ER binding (e.g. tamoxifen) or at blocking estrogen biosynthesis and preventing ER activation (aromatase inhibitors – AIs) – remains the mainstay for the treatment of ERα-positive disease. Despite its considerable initial efficacy, the development of resistance (seen in approximately 50% of patients) has been a major hurdle to curative BC treatment. Few efficacious treatment options are available for the treatment of resistant disease, and hence, the discovery of new therapies is highly desirable.

Apoptosis is a process of programmed cell death crucial for development, normal cell turnover and appropriate immune response (Green 2019). Apoptosis can occur via the intrinsic or extrinsic pathway, which converges to activate effector cysteine-aspartate proteases (caspases) responsible for initiating protein and DNA degradation, a hallmark of apoptosis (Carneiro & El-Deiry 2020*a*). Initiator caspases (caspase-2, -8, -9, -10) and effector caspases (caspase-3, -6, -7) are synthesised as inactive precursor forms (Green 2022*a*). The extrinsic pathway is initiated by the binding of death-inducing ligands such as FasL to a death receptor (FasR) on the cell surface (Carneiro & El-Deiry 2020*a*, Green 2022*a*). This promotes the formation of a death-inducing signalling complex and the cleavage and activation of caspase-8. The intrinsic pathway is activated when pro-apoptotic molecules (e.g. cytochrome c, second mitochondrial activator of caspases (Smac)), which are normally retained in the mitochondria, are released into the cytoplasm following disruption of mitochondrial membrane integrity. These activate caspase-9, and subsequently caspases (Green 2022*a*). The B-cell lymphoma 2 (Bcl2) – family of proteins consisting of pro- (Bax, Bad) and anti-apoptotic members (Bcl2, Bcl-XL) – can alter mitochondrial membrane permeability depending on their cellular expression (Green 2022*b*). Apoptosis is a tightly regulated process and an important mechanism of eliminating damaged cells to maintain the integrity of the system/organism. As resistance to therapy involves bypassing apoptosis, identification of apoptosis-inducing targets may be a promising approach for the treatment of therapy-resistant BC.

microRNAs (miRs) are 21–23 nt non-coding RNAs that regulate target genes primarily by binding to 3’ untranslated regions (3’UTRs) of mRNAs and inhibiting translation, most frequently resulting in transcript degradation (Jonas & Izaurralde 2015). miRs represent an important layer of regulation of gene expression. They have been implicated in tumourigenesis and can regulate tumour progression, metastasis and drug resistance (Adams *et al.* 2014). Their importance in cancer progression supports the modulation of their function or abundance for the treatment of therapy-resistant BC (van Schooneveld *et al.* 2015). miRs are transcribed as primary miRs (pri-miR), which are cleaved and processed by an RNase III enzyme, Drosha and its cofactor, DiGeorge Critical Region 8 (DGCR8), producing a ~64 nt precursor miR (pre-miR) (Treiber *et al.* 2019). Following Exportin-5-mediated nuclear export, the pre-miR is cleaved by Dicer to produce a ~22 nt dsRNA duplex (Kim *et al.* 2016). The miR duplex is recruited to the RNA-inducing silencing complex (RISC), containing helicases and endonucleases (Argonaute, AGO) (O'Brien *et al.* 2018). Upon degradation of the ‘passenger’ strand, RISC is ‘guided’ by the remaining strand to the target mRNAs (O'Brien *et al.* 2018). miRs are commonly dysregulated in therapy-resistant BCa (Iorio *et al.* 2005, Yerukala Sathipati & Ho 2018), where they can also regulate apoptosis (Breunig *et al.* 2017, He *et al.* 2019).

Taken together, these suggest that miRs may be exploited therapeutically to induce apoptosis in therapy-resistant BCa. miR-based therapies have many potential benefits over conventional treatments: their small size facilitates cell entry, they are highly stable in blood, they demonstrate high target specificity and can be modified for tissue-specific delivery (Roberts *et al.* 2020).

We hypothesised that miRs can modulate apoptosis in BC and that apoptotic miRs may be altered upon therapy resistance onset. To this end, a high-throughput miR inhibitor screen was conducted in MCF7 BC cell line model and its AI-resistant (LTED) and tamoxifen-resistant (TamR) derivatives. Following miR inhibitor library transfection, screening microscopy was used to read out the fluorescent caspase-3/7 activity. We show that miR-361-3p inhibition induces apoptosis and inhibition of proliferation across drug-resistant and -responsive BC cell lines *in vitro, in vivo* and *ex vivo* through p53-mediated G1 cell cycle arrest and in part through FANCA modulation. miR-361-3p expression is associated with reduced BC patient survival and its inhibitors may represent effective therapeutics for BC treatment, both in endocrine-responsive and -resistant disease, particularly since miR-361-3p inhibitor showed synergy with PARP inhibition.

## Materials and methods

### Cell culture

MDA-MB-231, T47D and HEK293 were maintained in Dulbecco’s-Modified Eagle’s Medium (DMEM) supplemented with 10% fetal calf serum (FCS) and 2 mM l-glutamine (complete growth medium). Parental MCF7 were maintained as mentioned earlier but with addition of 10 nM estradiol. Tamoxifen-resistant (TamR) and fulvestrant-resistant (FulvR) MCF7 derivatives were maintained in complete growth medium with addition of 100 nM tamoxifen or fulvestrant, respectively. The long-term estrogen deprived (LTED) MCF7 derivative line was maintained in phenol red-free DMEM supplemented with 10% charcoal-dextran-stripped FCS (csFCS) and 1% l-glutamine. MCF7, TamR and LTED cells were a kind gift from Dr Luca Magnani (Imperial College London, UK) and FulvR, MDA-MB-231 and T47D were kindly provided by Prof Simak Ali (Imperial College London, UK). All cell lines were kept at 37°C in a humidified atmosphere with 5% CO_2_.

### High-throughput microRNA inhibitor screening

High-throughput screening was performed in MCF7, TamR and LTED cells using miRCURY LNA miRNA Inhibitor Library (Qiagen) at 20 nM. The library consists of 954 antisense oligonucleotides with sequences perfectly complementary to their respective miR target. Also included were mock transfection, LNA negative control A (Qiagen), AllStars Hs Cell Death Control siRNA (Qiagen) and AllStars Negative Control siRNA (Qiagen). Cells were seeded at 1800 cells/well (TamR, LTED) and 1500 cells/well (MCF7) in 384-well black-walled plates in 40 µL phenol red-free DMEM containing 10% charcoal-stripped FCS. After 24 h, 10 µL of CellEventTM Caspase-3/7 Green 74 Detection Reagent (Invitrogen) diluted to 12 µM in the medium was added (final concentration after addition of transfection complexes: 2 µM). A total of 10 µL transfection complex was then added per well, consisting of lipofectamine RNAiMax reagent (0.075 µL; Invitrogen), miR inhibitor from the library (3.6 µL at 333 nM) and OptiMEM (6.325µL), giving a final well volume of 60 µL. Cells were incubated for 72 h at 37°C and 5% CO_2_ and fixed by removal of 30 µL medium and addition of 30 µL of 6% paraformaldehyde in PBS for 20 min at room temperature. Cells were washed twice with PBS, removing 45 µL of final PBS wash prior to the addition of 45 µL 1/4000 Hoechst (1/5000 final concentration). Cells were incubated for 30 min and washed once with PBS. Plates were stored at 4°C in 50:50 PBS:glycerol. Caspase 3/7 and Hoechst fluorescent signal was quantified using a CellInsight™ CX5 High Content Screening microscope (ThermoFischer Scientific™). Caspase 3/7 signal was quantified in the nucleus only and the percentage (0–1.00) of dying cells per field of view was calculated, using Hoechst fluorescence to quantify cell number. This percentage ranging from 0 to 1.00 is termed apoptotic score.

### Data analysis and processing of miR inhibitor screening data

Log fold change (LFC) of individual miR inhibitors were calculated as LFC = log(𝑋̅*_resistant_*/MCF7), where 𝑋̅*_resistant_* is the mean of the apoptotic score in LTED and TamR, and MCF7 is the score in MCF7. In addition, robust Z-scores of the LFC of each individual miR inhibitor were calculated. The median of the LFC of all 954 miR-inhibitors (*xmedian* = 0.52) was determined in order to calculate median absolute deviation (MAD) as *MAD* = media (|*x_i_* − *xmedian*|), where *xi* is the LFC of each miR-inhibitor and *xmedian* is the mean LFC of all inhibitors. With MAD (=0.31), the robust Z-score of the LFC (RZ-score(LFC)) of each miR inhibitor was calculated as *RZscore_i_* = (0.6745 (*x_i_* − *xmedian*))/*MAD*, where *i* corresponds to each individual miR inhibitor LFC. The 0.6745 value is obtained from the 0.75th quartile of the standard normal distribution table, to which the MAD converges. Using the standard normal distribution table as a reference, miR-inhibitors with an absolute RZ-score(LFC) ≥ 2.5 have a significantly different LFC with *P* ≤ 0.00621.

## Lipofectamine RNAiMax-mediated oligonucleotide transfections

Transfection was performed using lipofectamine RNAiMax (Invitrogen by ThermoFisher Scientific™) according to the manufacturer’s protocol.

### Unassisted oligonucleotide transfections

Antisense LNA miR inhibitors (Qiagen) – ASO-361-3p and ASO-NC – were added directly to cell culture medium to final concentrations of 20 nM to 2 µM.

### Plasmid DNA transfection

Cells seeded in six-well plates were transfected with plasmid DNA (0–2 µg) using JetPrime transfection reagent (Polyplus) according to the manufacturer’s instructions. The medium was changed 4–6 h post-transfection.

### Sulphorhodamine B cell growth assay

Cells seeded at 4000 cells/well in 96-well plates in either complete growth medium (MCF7 and TamR) or phenol red-free DMEM containing 5–10% csFCS (LTED) for 24 h were transfected with miR inhibitors and/or mimics (0–30 nM) ± Olaparib (0–8 µM) or equal volume of DMSO vehicle. Cells were fixed at day 0, 3 and 6 by addition of equal volume of 40% TCA for 1 h at 4°C prior to rinsing in tap water and air drying. Fixed cells were stained by addition of 0.04% (w/v) sulphorhodamine B (SRB) in acetic acid for 1 h at room temperature. Plates were rinsed ×5 with 1% (v/v) acetic acid five times and air dried. SRB was reconstituted with 10 mM Tris-HCl and the optical density was measured at 492 nm with a Sunrise™ absorbance reader (Tecan, Männedorf, Switzerland).

### Caspase 3/7-Glo assays

Cells were seeded into white-walled, clear-bottomed 96-well plates (6000 cells/wells) and treated as described for SRB growth assay for 72 h. Caspase-3/7-Glo assay (Promega) was performed according to the manufacturer’s instructions. Caspase activity was normalised to cell number by SRB assay. Note that, although MCF7 cell line and its derivatives do not express caspase-3, apoptosis can proceed via caspase-7.

### Flow cytometric analysis of early and late apoptosis

MCF7, TamR, LTED, FulvR and MDA-MB-231 cells transfected with miR mimics and/or inhibitors as described earlier (0–30 nM) for 72 h were prepared for flow cytometry using the annexin V staining assay for flow cytometry (ThermoFisher Scientific™) according to the manufacturer’s protocol. Annexin V-positive, propidium iodide (PI)-negative cells represent early apoptosis, late apoptotic cells are positive for both annexin V and PI, and necrotic cells are annexin V-negative but PI-positive.

### SDS-PAGE and Western blotting

Whole cell lysates prepared in RIPA buffer supplemented with protease and phosphatase inhibitors were resolved on 8–15% SDS-PAGE gels and electroblotted onto PVDF membrane. Membranes were blocked with 5% skimmed milk powder prepared in PBST (0.05% Tween-20 in phosphate buffered saline), or 5% bovine serum albumin in TBST (0.05% Tween-20 in Tris-buffered saline) and incubated in primary antibody prepared in blocking buffer: anti-PARP1 (Cell Signaling, #9542), anti-cleaved PARP1 (Cell Signaling #9541), anti-β-actin (Abcam ab8227), anti-FANCA (Abcam ab201457), anti-Rb (Abcam ab6075-1), anti-phospho-Rb (Ser807) (Cell Signaling #8516), anti-p53 (Santa Cruz Biotechnology, sc126). Membranes were washed with PBST or TBST and incubated with HRP (horse radish peroxidase)-conjugated secondary antibodies and blots were developed with Luminata™ forte (Merck-Millipore) and imaged using iBright (Invitrogen). Densitometry was performed using ImageJ.

### Apoptosis antibody arrays

Protein lysates were prepared to 500 µg/mL and antibody array analysis was performed using Human Apoptosis Antibody Array (Abcam ab134001) according to the manufacturer’s protocol. Membranes were imaged using the Fusion Solo Chemiluminescence Imager. Individual spots were quantified using ImageJ, with the background (negative control spots) subtracted from each sample and normalised against its own positive control (biotin-conjugated IgG) from the same membrane.

### RNA isolation

Total RNA was isolated from cell lines and flash-frozen tissues using the Monarch Total RNA Miniprep Kit according to the manufacturer’s protocol. For tissue, the flash-frozen mouse tissue or MCF7 xenograft tumours were homogenised using the Precellys 24 Tissue Homogeniser (Bertin Instruments) for two cycles of 25 s at 4500 ***g*** in 500 µL of 1× DNA/RNA Protection Reagent from the Monarch Total RNA Miniprep Kit.

### MicroRNA-specific reverse transcription and qPCR

Of the total RNA, 10–20 ng were reverse-transcribed using the miRCURY LNA RT Kit (Qiagen), cDNA was diluted 1:15–1:30 and qPCR was performed using miRCURY SYBR Green PCR Master Mix (Qiagen) and miRCURY LNA miRNA PCR assays (Qiagen) according to the manufacturer’s instructions. Data analysis was performed with the ΔΔCt method, using hsa-Snord48 and U6 for normalisation.

### RNA reverse transcription and qPCR

Total RNA was isolated from cell lines or tissues as described earlier, and 405 ng were reverse transcribed using the Precision nanoScriptTM 2 Reverse Transcription Kit (Primer Design). cDNA was diluted 1:5 and 2 µL were used in a 10 µL qPCR reaction, using 2× SYBR Green Fast Master Mix (Life Technologies) and 400 nM forward and reverse primers. Reactions were cycled on QuantStudio 7 and analysed using the ΔΔCt method, with L-19 and GAPDH used for normalisation.

### Immunofluorescent staining and microscopy

Cells seeded onto coverslips were fixed with 4% paraformaldehyde in PBS for 10 min at room temperature. Fixed cells were washed ×2 with PBS and permeabilised with 0.5% TritonX in PBS for 10 min at room temperature followed by blocking in 10% goat serum in PBS for ~3 h. Primary antibody (anti-phospho-γH2AX(Ser139) – Merck Millipore 05-656) diluted 1:1000 in 10% goat serum was added to fixed permeabilised cells overnight at 4°C, followed by PBS washing. Secondary antibody (AlexaFluor goat anti-mouse 488) diluted 1:200 in 10% goat serum was added to slides for 1 h at room temperature in the dark, washed with PBS and counterstained with DAPI (1 µg/µL) for 10 min at room temperature in the dark. After final PBS washing, coverslips were mounted onto glass slides in phalloidin-containing Vectashield mounting medium (Vector laboratories). Slides were imaged on Zeiss Meta 512 confocal microscope.

### Invasion assays in gelatin-coated transwells

Cells in suspension were incubated with calcein AM (Abcam) (1 µg/mlL for 30 min at room temperature in the dark. A total of 3000 cells were seeded onto transwells coated with 2.5% gelatin and incubated at 37°C for 24 h. Gelatin-coated transwells were prepared as previously described (Culig 2018). Invading cells were imaged using the EVOS cell imaging system (ThermoFisher Scientific™).

### MCF7 xenograft experiments

MCF7 xenografts were established on right flanks of 6-week-old female BALB/c nude mice (Envigo) by injection of 5 × 10^6^ MCF7 cells resuspended in 100 µL PBS under 3% isoflurane anaesthesia (Abbott Animal Health UK). After recovery in a heat chamber, mice were returned to cages with access to drinking water containing 8 µg/mL estrogen *ad libitum*. At tumour volume = 100 mm^3^, mice were randomly assigned to a vehicle (PBS), negative control LNA anti-sense oligonucleotide (ASO-NC) or hsa-miR-361-3p-V1 anti-sense oligonucleotide (ASO-361-3p), *n* = 6/group (PBS, ASO-NC) or *n* = 7/group (ASO-361-3p). ASOs and vehicles were administered by tail vein injection at a dose of 10 mg/kg twice weekly for 18 days. Tumour volume was calculated five times a week as (width^2^/(length/2)). Mice received water supplemented with estrogen (8 µg/mL) and food *ad libitum* and were monitored daily for ill effects. All procedures were conducted in accordance with regulations under the Animal (Scientific Procedures) Act 1986 of the United Kingdom (HMSO, London, UK, 1990) and with appropriate local ethical and Health and Safety approval. At the experiment end, mice were euthanised and tumours resected. Half of the tumour was flash-frozen for RNA/protein isolation and half formalin-fixed for immunohistochemistry. Brain, heart, lung, liver, spleen, kidney, muscle and bone were also harvested as mentioned earlier for RNA isolation.

### Immunohistochemistry

Immunohistochemistry was performed as described (Leach *et al.* 2021) (see also Supplementary information, see section on supplementary materials given at the end of this article).

### RNA-sequencing of ASO-361-3p-treated MCF7 xenografts

Five nanograms of total RNA extracted from six MCF7 tumour xenografts (*n* = 3 ASO-NC-treated, *n* = 3 ASO-361-3p-treated) were used to prepare cDNA libraries using the TruSeq® Stranded mRNA Library preparation kit and the TruSeq® RNA Single Indexes Set A (Illumina Inc., San Diego, California, USA), according to the supplier’s instructions. Samples were quantified with the QubitTM DNA High Sensitivity Assay Kit (ThermoFisher Scientific) in a Qubit® 4 Fluorometer (ThermoFisher Scientific™). Library quality and concentration were assessed using the High Sensitivity DNA D1000 ScreenTape® Analysis kit (Agilent Technologies) in an Agilent 2200 TapeStation System (Agilent Technologies) as specified by the supplier. Paired-end sequence reads of 100 base pairs long were produced using a HiSeq2500 instrument (Illumina).

### Bioinformatic processing of RNA-sequencing data

Sequencing quality was assessed using FastQC software version 0.11.8 (Babraham Bioinformatics Institute). Libraries were aligned to UCSC hg19 genome using HiSat2 2.1.0 (Kim *et al.* 2016). Data analysis and visualisation were performed in R version 3.6.1 programming environment. Aligned sam files were imported into R and converted into bam files with the Rsamtools R package. The GenomicFeatures R package was used to retrieve genomic locations of exons from the UCSC platform in a TxDb object, and read counts were generated with the SummarizeOverlaps function of the R package GenomicAlignments v1.8.4 (Lawrence *et al.* 2013). The R package edgeR was used to filter low-expressed genes, normalise the reads by the trimmed mean of M-values (TMM) method and determine differentially expressed genes (DEGs) (Robinson *et al.* 2010). Volcano plots were generated with the ggplot2 and tidyverse R packages. Venn diagrams were drawn with the VennDiagram package, and principal component analysis (PCA) plots were generated with the pcaMethods R package. Finally, gene set enrichment analysis (GSEA) was performed using the Molecular Signatures Database (MSigDB) ‘Hallmark’ gene set collection (Liberzon *et al.* 2015).

### Analysis of TCGA and METABRIC small RNA-seq data

In the TCGA-BRCA dataset, matched normal adjacent and primary tumour tissue small RNA-seq data were available for 110 invasive ductal carcinoma (IDC) BC patients, and matched metastatic tumour and primary tumour tissue small RNA-seq data were available for 7 patients. Clinicopathological values such as the molecular BC subtype were obtained from Liu *et al.* (2018). The normalised miR expression levels for TCGA-BRCA were downloaded using the *TCGAbiolinks* R package. In addition, normalised miRNA gene expression from the Molecular Taxonomy and Breast Cancer International Consortium (METABRIC) cohort was downloaded with pyega3 and the aspera system. A total of 86 paired normal adjacent and primary IDC BC tumours were analysed. Mature miR-361-3p expression was examined in the abovementioned samples using R version 3.6.1. Graphs were produced with ggplot2 R package. The Wilcoxon test was used to assess the statistical significance of differences in miR levels between adjacent normal and primary BC tissues, as well as between primary and metastatic tumour tissue. The Kruskal–Wallis test was used to assess the relationship between miR-361-3p expression and BC molecular subtypes. Correlations were calculated using Pearson’s correlation coefficient. Two-sided *P*-values < 0.05 were considered statistically significant for all tests.

### Breast cancer explant culture and anti-sense oligonucleotide treatment

Freshly resected BC tumour specimens were obtained from patients undergoing surgery at Charing Cross Hospital (London, UK). The malignant region of tissue was resected using an automatic biopsy system (CareFusion) or scalpel and transported to the laboratory within 2 h of surgery on ice in RPMI media supplemented with 10% FCS, 1% penicillin/streptomycin/glutamine, 0.01 mg/mL hydrocortisone and 0.01 mg/mL insulin. The patient-derived explant *ex vivo* culture method is based on that described by Centenera *et al.* (2013). Briefly, under sterile conditions, tumour specimens were PBS-washed and diced into 1 mm^3^ pieces using a scalpel. In parallel, ASO-NC and ASO-361-3p were prepared to 20 µg/mL in the above culture medium to mirror dosing used in the xenograft studies. One millilitre of diluted ASO solution was added to Surgispon® absorbable gelatin sponge (Aegis Lifesciences, Ahmedabad, Gujarat, India) and allowed to soak at room temperature for 15 min during explant preparation. ASO medium-soaked gelatin sponges were transferred to 24-well plates and an additional 500 μL ASO-containing medium were added to each well. Four 1 mm^3^ explants were placed on top of each sponge and incubated at 37°C for 48 h. Half of explants were flash-frozen for RNA extraction, and half were formalin-fixed for paraffin-embedding and immunohistochemistry.

### Statistical analysis

Normally distributed, continuous variables were assessed by Student’s *t*-test. *P* ≤ 0.05 was interpreted to denote statistical significance. Results are presented as mean ± s.e. or ± s.d. for at least three independent experiments unless otherwise stated. Pearson correlation coefficient was calculated assuming a linear relationship between variables.

## Results

**Figure 1:**
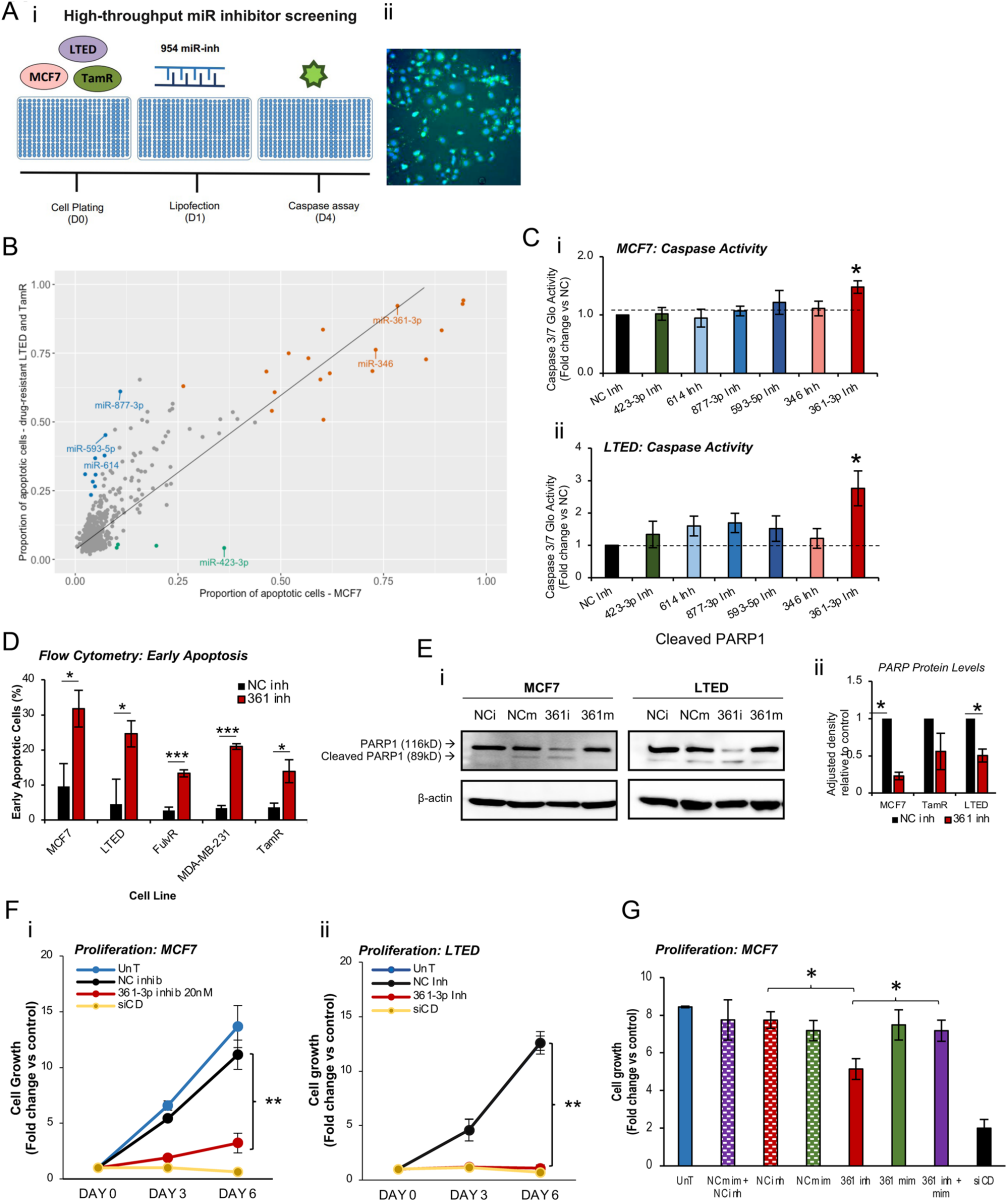
miR-361-3p inhibition induces early apoptosis and represses proliferation in drug-responsive and -resistant breast cancer cells. (A) Workflow of high-throughput miR inhibitor screening: a library of 954 miR inhibitors was transfected into MCF7, TamR and LTED breast cancer cells and high-content screening microscopy was conducted for CellEvent Apoptotic marker (green) and Hoechst nuclear stain (blue) 72 h post-transfection. The screening was performed in biological triplicate and an example image is shown in (ii). (B) Scatter graph illustrating mean proportion of apoptotic cells in endocrine therapy-resistant (TamR and LTED) cells (Y-axis) and parental MCF7 cells (*X*-axis) for each individual miR-inhibitor (dot). Coloured are the miR inhibitors that met criteria 1 (green, induce significant apoptosis in ≥10% of drug-responsive MCF7 cells only), criteria 2 (blue, induce significant apoptosis in ≥20% of resistant lines (LTED and TamR)) or criteria 3 (red, induce cell death in ≥50% of all three cell lines). Top six miRs selected for further investigation are labelled. C) Caspase 3/7-Glo Luciferase assay analysis of caspase-3/7-mediated apoptosis in MCF7 and LTED cells upon miR-346, -361-3p, -423-3p, -614, -593-5p and -877-3p inhibitor transfection (20 nM) normalised to cell number by sulforhodamine B (SRB) assay. Inhibitors are denoted as ‘Inh’. Columns: mean fold change ± s.e.m. in caspase 3/7 activity at day 3 relative to non-targeting negative control inhibitor for three independent experiments performed in triplicate (*p < 0.05). (D)Flow cytometric annexin-V/propidium iodide analysis of early apoptosis in MCF7, LTED, FulvR, MDA-MB-231 and TamR breast cancer cell lines upon miR-361-3p or NC inhibitor transfection for 72 h. Inhibitors were transfected to 20 nM. Columns: mean percentage of cells in annexin V-positive, PI-negative population (early apoptosis) for three independent experiments ± s.e.m. (E)Western blot analysis of PARP1 protein levels in MCF7 and LTED cells transfected with miR-361-3p inhibitor (20 nM), -mimic (30 nM) or controls for 72 h. β-actin was used as a loading control and densitometry (ii)was performed using ImageJ. Columns: mean full-length PARP1 band densities normalised to β-actin. (F,G)Sulphorhodamine B (SRB) assay analysis of (F)MCF7 (i) and LTED (ii) and (G)MCF7 cell proliferation following transfection with (F)negative control (NC) and miR-361-3p inhibitors (20 nM) or siCellDeath (siCD – 20 nM) for 6 days or (G)NC mimic, NC inhibitor, miR-361-3p inhibitor and miR-361-3p mimic or indicated combinations (10 nM) for 3 days. (F,G)Points/columns represent mean relative cell growth (mean absorbance per condition at day 3 or 6 relative to day 0 absorbance) ± s.e.m. siCD targets essential viability transcripts and is a positive control for growth inhibition. Inhibitors are denoted as ‘inh’, and mimics as ‘mim’, respectively. Mock transfection is denoted as ‘UnT’. (*P ≤ 0.05, **P ≤ 0.005, ***P ≤ 0.0001).

miRs have been implicated in a wide array of diverse cellular processes, including apoptosis. However, their differential contributions to apoptotic signalling in the context of drug-responsive and drug-resistant BC have not been systematically explored. To this end, we performed high-throughput miR inhibitor screening in MCF7 cells and their AI- and tamoxifen-resistant derivatives, using high-content screening microscopy to quantify caspase 3/7-mediated apoptosis and cell number (Fig. 1Ai). An example image is shown (Fig. 1Aii). The number of apoptotic cells per field across five fields was quantified per inhibitor. This identified 21 miR inhibitors that significantly induced apoptosis in one or more of the BC cell lines at Z-score > ±2.5, *P* ≤ 0.006. To select a panel of miR inhibitors for *in vitro* validation, the inhibitors were separated into three categories: i) miR inhibitors increasing apoptosis in hormone-responsive, but not -resistant lines (*n* = 4, green; Fig. 1B, ii) inhibitors inducing apoptosis in hormone-resistant but not -responsive cells (*n* = 9, blue; Fig 1B) and iii) inhibitors increasing apoptosis in drug-responsive and one or both of the drug-resistant BC cell lines (*n* = 13, red; Fig. 1B). Supplementary Tables 1, 2 and 3 detail the individual miRs identified in each category. Six miRs were selected for validation: miR-423-3p (increased apoptosis in MCF7 only); miR-614, miR-877-3p and miR-593-3p (increased apoptosis in one or both of drug-resistant lines only) and miR-346 and miR-361-3p (increased apoptosis in all three BC cell lines). Caspase 3/7-Glo assays revealed that only miR-361-3p inhibitor significantly increased caspase-mediated apoptosis in MCF7 and LTED, but not TamR cells (Fig. 1C, Supplementary Fig. 1), although we do not discount the possibility that these miR inhibitors may induce apoptosis by other pathways, particularly since caspase-3 is not present in MCF7 cells. qPCR confirmed that miR-361-3p inhibitor reduces endogenous miR-361-3p levels upon transfection into BC cell lines (Supplementary Fig. 2).

To examine miR-361-3p inhibitor-induced apoptosis in greater detail, flow cytometry was performed on cells stained with annexin-V and PI. Annexin-V-positive, PI-negative cells represent those in the early stages of apoptosis, whilst during late-stage apoptosis, loss of membrane integrity allows additional uptake of PI. It was shown that miR-361-3p inhibitor significantly increased the percentage of early apoptotic cells in all of MCF7, LTED, FulvR, MDA-MB-231 and TamR cells (Fig. 1D). Further, miR-361-3p inhibition significantly reduced full-length PARP protein levels in MCF7 and LTED, but not TamR cells (Fig. 1E, Supplementary Fig. 3), indicative of PARP cleavage during apoptotic induction and potential differences in mechanisms of apoptotic induction in response to miR-361-3p inhibition in TamR vs MCF7 and LTED. To assess whether miR-361-3p inhibitor-mediated apoptosis is translated to an inhibitory effect on BC cells proliferation, SRB assays were performed on MCF7 and LTED cells following transfection with miR-361-3p or NC inhibitor, or siCD (positive control for inhibition of proliferation). miR-361-3p inhibition was found to significantly repress the growth of both MCF7 and LTED cells compared to NC and untreated cells, with effects comparable to siCD positive control (Fig. 1F). miR-361-3p mimic did not alter BC cell growth alone, likely due to optimal cell proliferation under complete medium conditions (Supplementary Fig. 4), but did rescue miR-361-3p inhibitor-induced growth inhibition (Fig. 1G). Of other candidate apoptosis-modulating miRs, miR-423-3p modulation did not significantly alter the proliferation of any BC cell line (Supplementary Fig. 5). miR-593-3p inhibitor significantly reduced proliferation of LTED cells, whilst no effect was observed with the mimic (Supplementary Fig. 6), with similar effects observed for miR-346 inhibitor (Supplementary Fig. 7).

**Figure 2:**
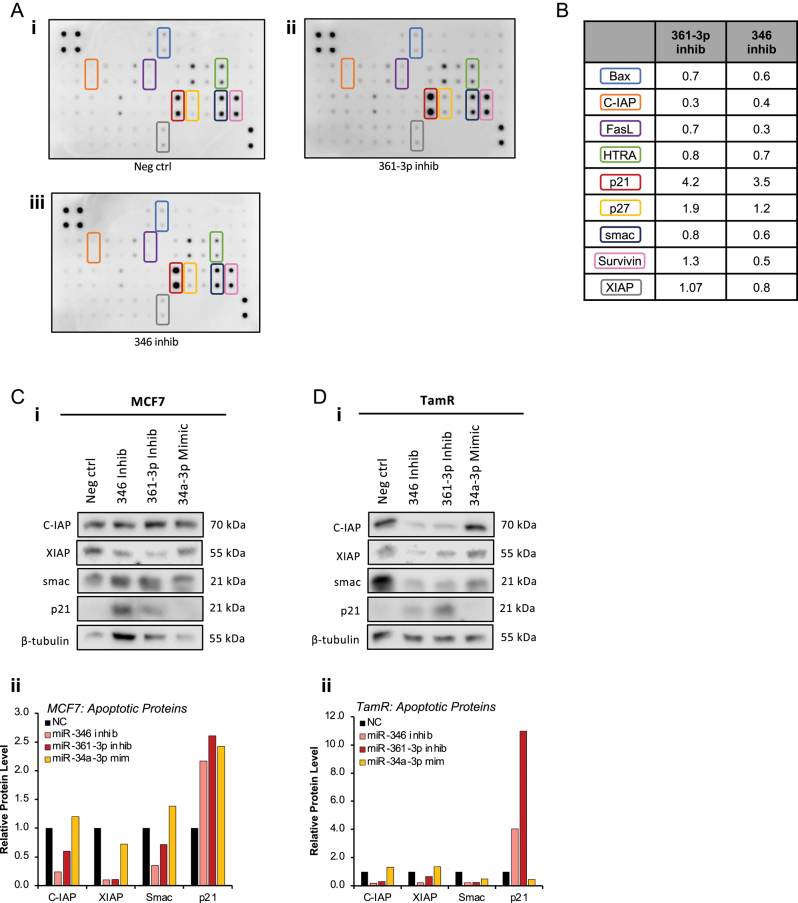
Apoptosis antibody array identifies apoptotic proteins mediating miR-361-3p inhibitor effects. (A,B) Apoptosis antibody array analysis of MCF7 cells transfected with NC miR inhibitor, miR-361-3p inhibitor or miR-346 inhibitor (20 nM) for 72 h. The table shows relative protein levels relative to negative control, and coloured boxes denote indicated proteins on array images. (C,D)Western blot assay analysis of (C)MCF7 and (D)TamR cells following transfection with miR-346 inhibitor, miR-361-3p inhibitor or miR-34a-3p mimic (20 nM) for 72 h. β-tubulin is used as a loading control and densitometry (ii)was performed using ImageJ. Columns: mean protein band densities normalised to β-tubulin.

To identify apoptotic proteins involved in miR-361-3p inhibitor-mediated apoptosis, antibody arrays were performed in MCF7 cells following transfection with NC, miR-361-3p or miR-346 inhibitor. It was shown that miR-361-3p inhibitor reduced protein levels of Bax, CIAP and Smac, whilst increasing p21 and p27, compared to NC-transfected cells (Fig. 2A and B). miR-346 inhibition similarly decreased Bax, CIAP, FasL and Survivin and increased p21 and p27 compared to NC-transfected cells (Fig. 2C). Western blotting was performed to validate antibody array findings, confirming that miR-361-3p and miR-346 inhibitor transfection reduced XIAP levels in MCF7 cells, and CIAP, XIAP and Smac protein levels in TamR cells vsNC. Both inhibitors were shown to increase p21 protein levels, with greater effect observed in TamR cells.

**Figure 3:**
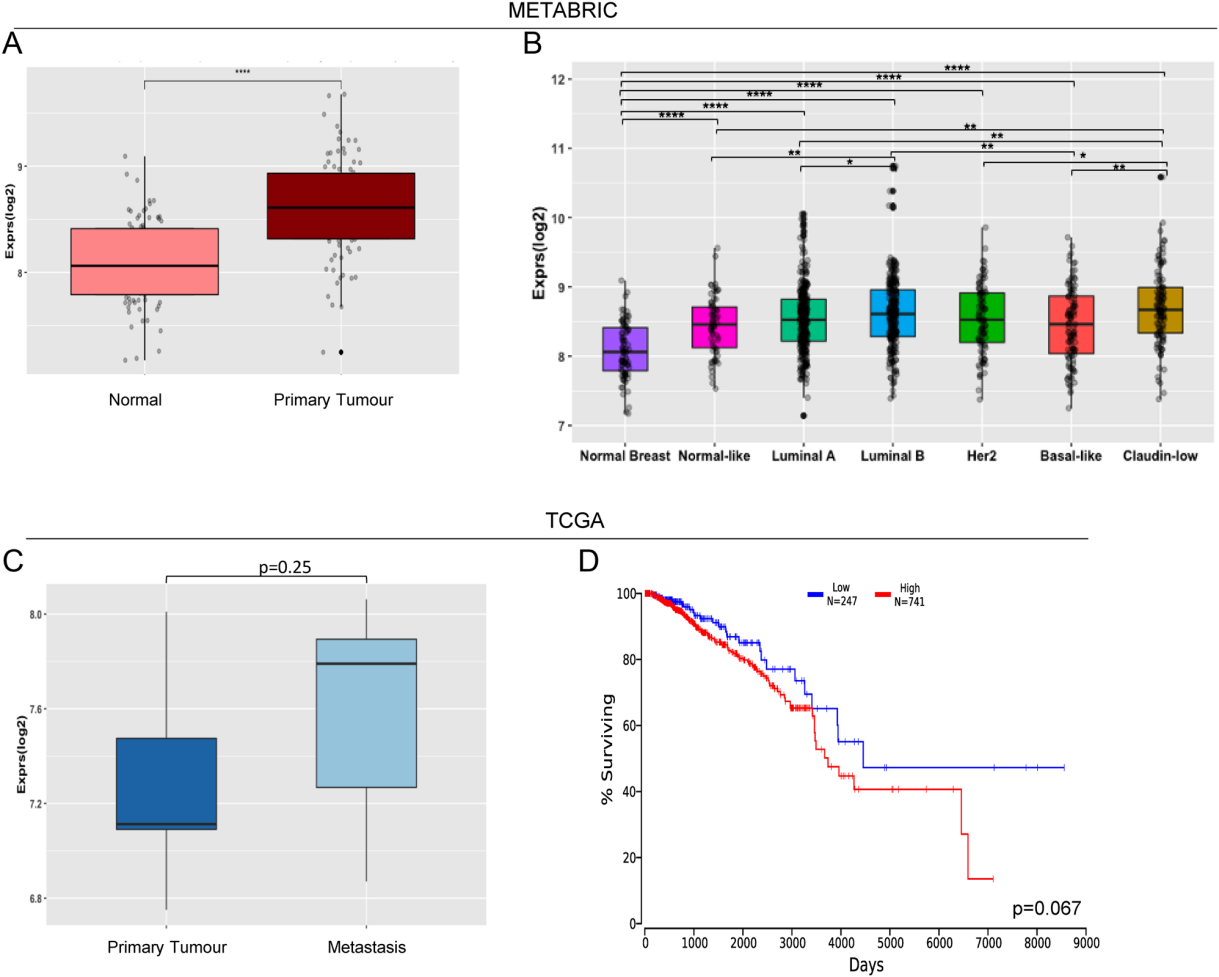
miR-361-3p is associated with enhanced breast cancer progression and poor disease outcome. (A)miR-361-3p expression in adjacent normal and primary tumour tissue of IDC BC patients of the METABRIC dataset. (B)miR-361-3p expression in normal breast and in IDC primary tumour samples divided by intrinsic molecular subtype (METABRIC dataset). Normalised data were downloaded from the METABRIC database and analysed with R software (packages ggplot2 and ggpubr). (C)miR-361-3p expression in primary and metastatic tumour tissues of seven IDC BC patients of the TCGA cohort. (D)Kaplan–Meier analysis of survival of patients from the TCGA-BRCA patient cohort stratified by miR-361-3p expression, showing improved survival of patients with low miR-361-3p compared to high miR-361-3p expression. The analysis was performed in http://www.oncolnc.org/ comparing survival of patients with high (top 75%) and low (bottom 25%) tumoural miR-361-3p. *P ≤0.05, **P ≤0.01, ***P ≤0.0001.

To confirm the relevance of miR-361-3p to clinical BC, we examined miR-361-3p expression in the METABRIC and TCGA-BRCA patient data sets. It was found that miR-361-3p levels were increased in primary tumour tissue as compared to normal adjacent tissues (Fig. 3A), and across all BC sub-types (Fig. 3B) in the METABRIC dataset. In TCGA-BRCA, miR-361-3p levels were non-significantly elevated in metastatic vsprimary tumour tissue (Fig. 3C). High miR-361-3p levels were associated with shorter patient survival (Fig. 3D), albeit non-significantly (*P* = 0.067). Importantly, miR-361-3p is well-expressed in drug-responsive and -resistant BC cell lines modelling different disease subtypes (Supplementary Fig. 8) and shows the highest expression levels of all candidate apoptosis-modulating miRs in patient tumour samples from TCGA (Supplementary Fig. 9A) and METABRIC (Supplementary Fig. 9B).

**Figure 4:**
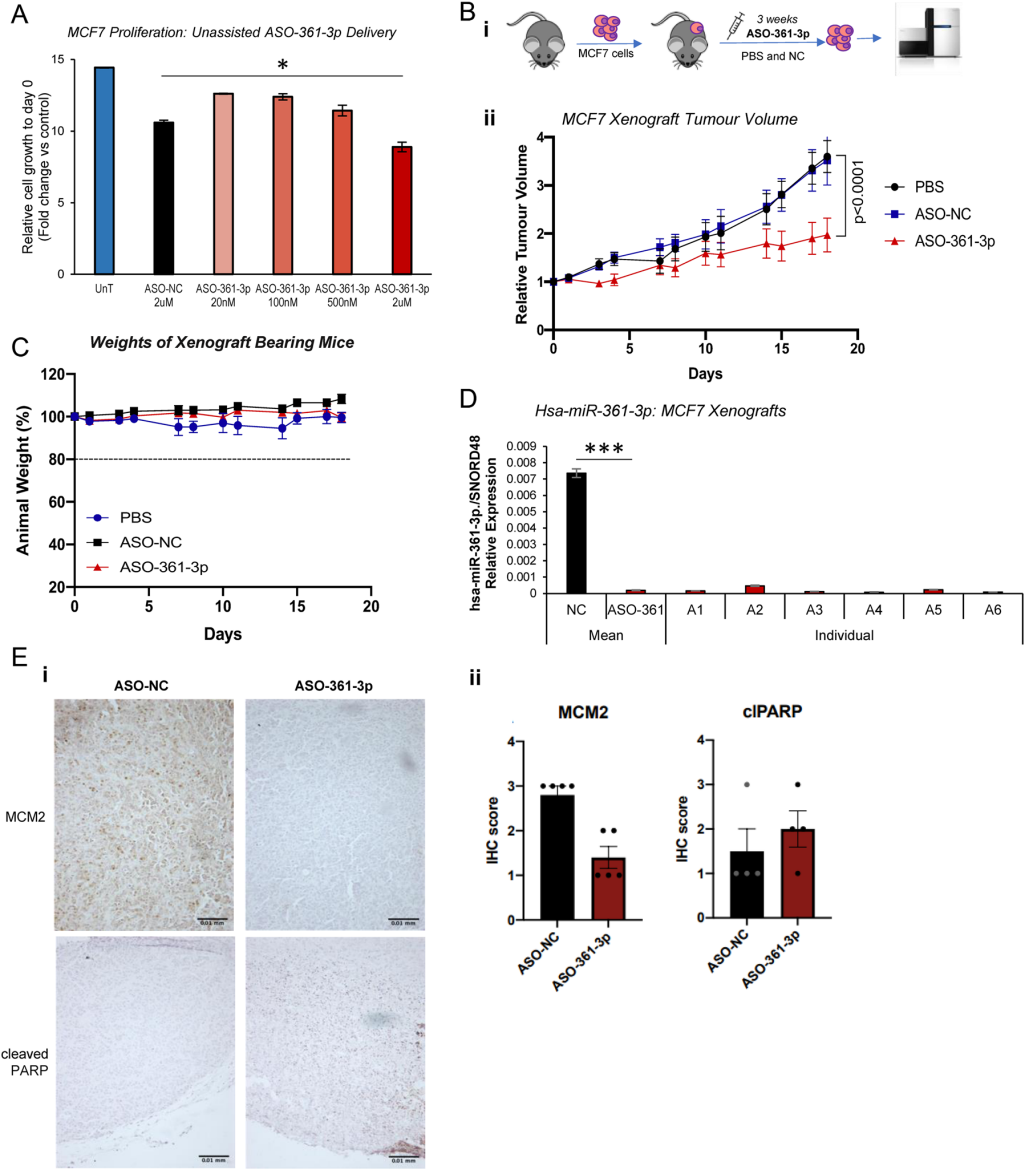
Inhibition of miR-361-3p using an anti-sense oligonucleotide inhibits breast cancer xenograft tumour growth *in vivo. (*A)SRB assay analysis of MCF7 cells proliferation following unassisted delivery of miR-361-3p anti-sense oligonucleotide optimised for *in vivo* use (ASO)-361-3p or NC (ASO-NC) for 6 days (20 nM–2 µM). Columns: relative cell growth (mean absorbance per condition at day 6 relative to day 0 absorbance) ± s.e.m. for three independent experiments performed in triplicate (B)Volume of MCF7 xenograft tumours following twice-weekly tail-vein injection of PBS, ASO-NC or ASO-361-3p (10 mg/kg for 13 days, 20 mg/kg for 5 days) into BALB/c nude mice for 18 days. Mean tumour volume per condition (volume = (width^2^) × (length/2) is shown and experimental plan illustrated. (C)Weights of BALB/c mice harbouring MCF7 xenograft tumours following twice-weekly tail-vein injection of PBS, ASO-NC or ASO-361-3p as above for 18 days. Mean percentage of day 0 weight is shown. (D)qRT-PCR analysis of hsa-miR-361-3p levels in MCF7 xenografts following treatment of host BALB/c mice as mentioned earlier for 18 days. Columns:mean endogenous hsa-miR-361-3p levels relative to Snord48 after 18 days treatment. (E)Immunohistochemical analysis of MCM4 (proliferation marker) and cleaved PARP (apoptosis marker) in sections of MCF7 xenograft tumours following treatment as mentioned earlier for 18 days. IHC was conducted on five tumours (MCM4) and four tumours (cleaved PARP). Brown stain denotes positivity. Staining intensity was scored (ii)according to the following criteria: 1 = weak intensity in <10% cells, 2 = weak staining in ≤20% of cells or moderate staining in ≤10%, 3 = moderate or strong staining in >10% cells (Garcia De La Torre *et al.* 2006). Representative sections are shown and additional sections shown in Supplementary Fig. 14. Columns: mean IHC scores ± s.e.m. *P ≤ 0.05, **P ≤ 0.01, ***P ≤ 0.0001.

We next sought to assess the impact of miR-361-3p inhibition on BC tumour growth *in vivo.* After confirming successful unassisted uptake of an *in vivo* use-modified miR-361-3p inhibitor (ASO-361-3p – sequence as Supplementary Fig. 10) by MCF7 cells, leading to growth suppression *in vitro* (Fig. 4A), MCF7 xenografts were established on the flanks of female Balb/c mice and PBS, ASO-NC or ASO-361-3p was delivered via twice-weekly tail vein injection for 18 days. ASO-361-3p was shown to significantly reduce BC xenograft tumour volume compared to ASO-NC- or PBS-injected mice (Fig. 4B, Supplementary Fig. 11). Importantly, ASO-361-3p-treated mice showed no difference in body weight compared to PBS- or ASO-NC-treated mice, indicating minimal systemic toxicity (Fig. 4C), and initial animal weights and tumour volumes showed no difference between treatment groups (Supplementary Fig. 12). qPCR analysis of hsa-miR-361-3p levels in xenograft tumours showed a robust reduction of miR-361-3p levels following ASO-361-3p treatment compared to ASO-NC treatment in all animals (Fig. 4D). Endogenous mmu-miR-361-3p levels were also significantly reduced in spleens, lungs and livers of ASO-361-3p- vsASO-NC-treated mice (Supplementary Fig. 13). Immunohistochemical staining of fixed tumour tissues revealed non-significantly reduced levels of proliferative marker, MCM2, in ASO-361-3p-treated tumours vscontrols, but no change in cleaved PARP protein levels (Fig. 4E, Supplementary Fig. 14).

**Figure 5:**
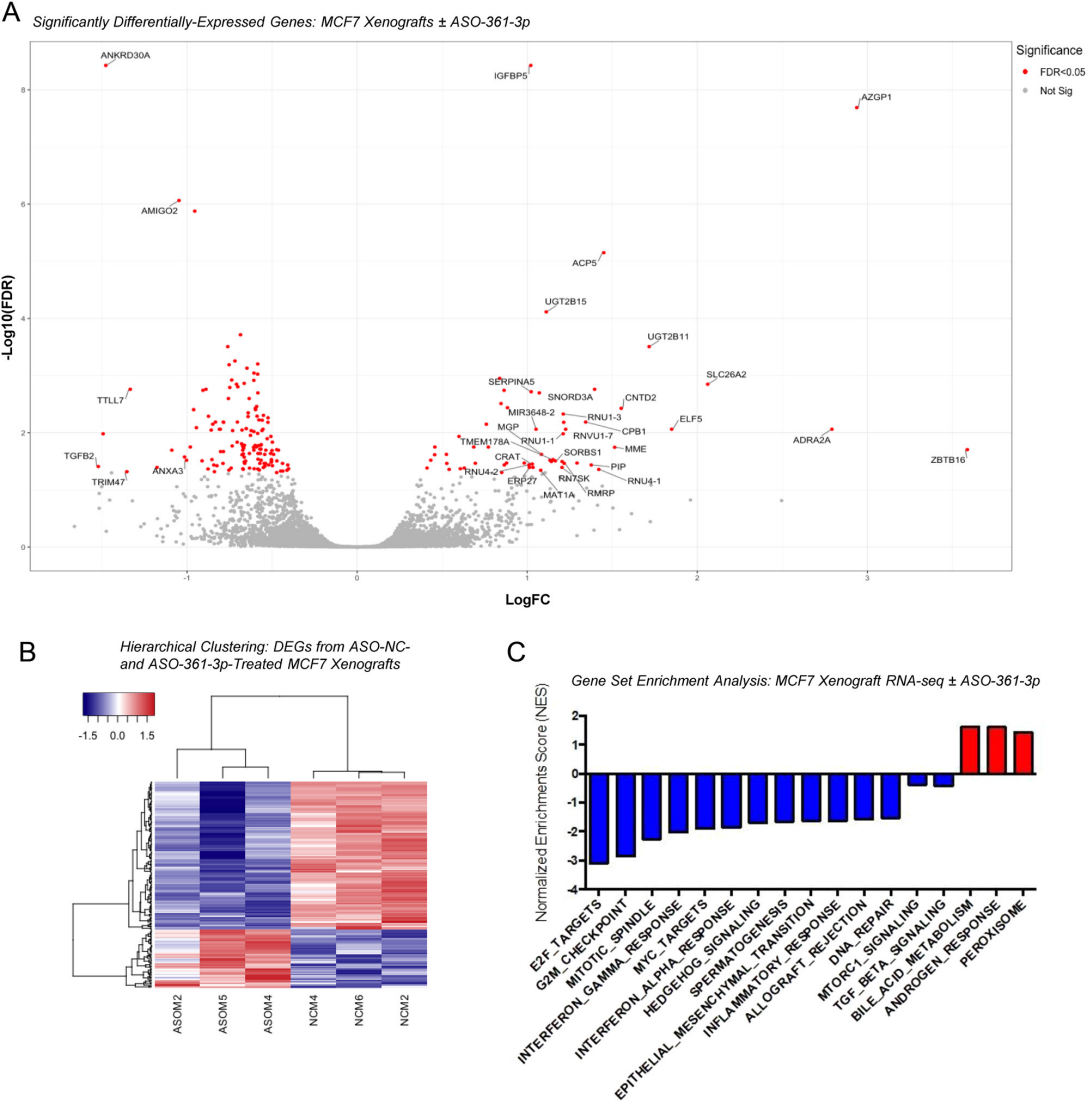
miR-361-3p inhibition modulates expression of cell cycle genes in breast cancer *in vivo*. (A)Volcano plot showing differentially expressed genes identified by RNA-seq of ASO-361-3p- vsASO-NC-treated breast cancer xenograft tumours. Red dots indicate 194 genes significantly differentially regulated (FDR < 0.05). Labelled genes are those significantly DE by more than ±1-fold. (B)Hierarchical clustering analysis of Z-score expression of the 194 genes differentially expressed following treatment of MCF7 xenografts with ASO-361-3p vsASO-NC for 18 days. (C)Gene Set Enrichment Analysis (GSEA) of the 194 genes differentially expressed following treatment of MCF7 xenografts with ASO-361-3p vsASO-NC for 18 days. Significant pathways (FDR < 0.25) are shown.

**Figure 6:**
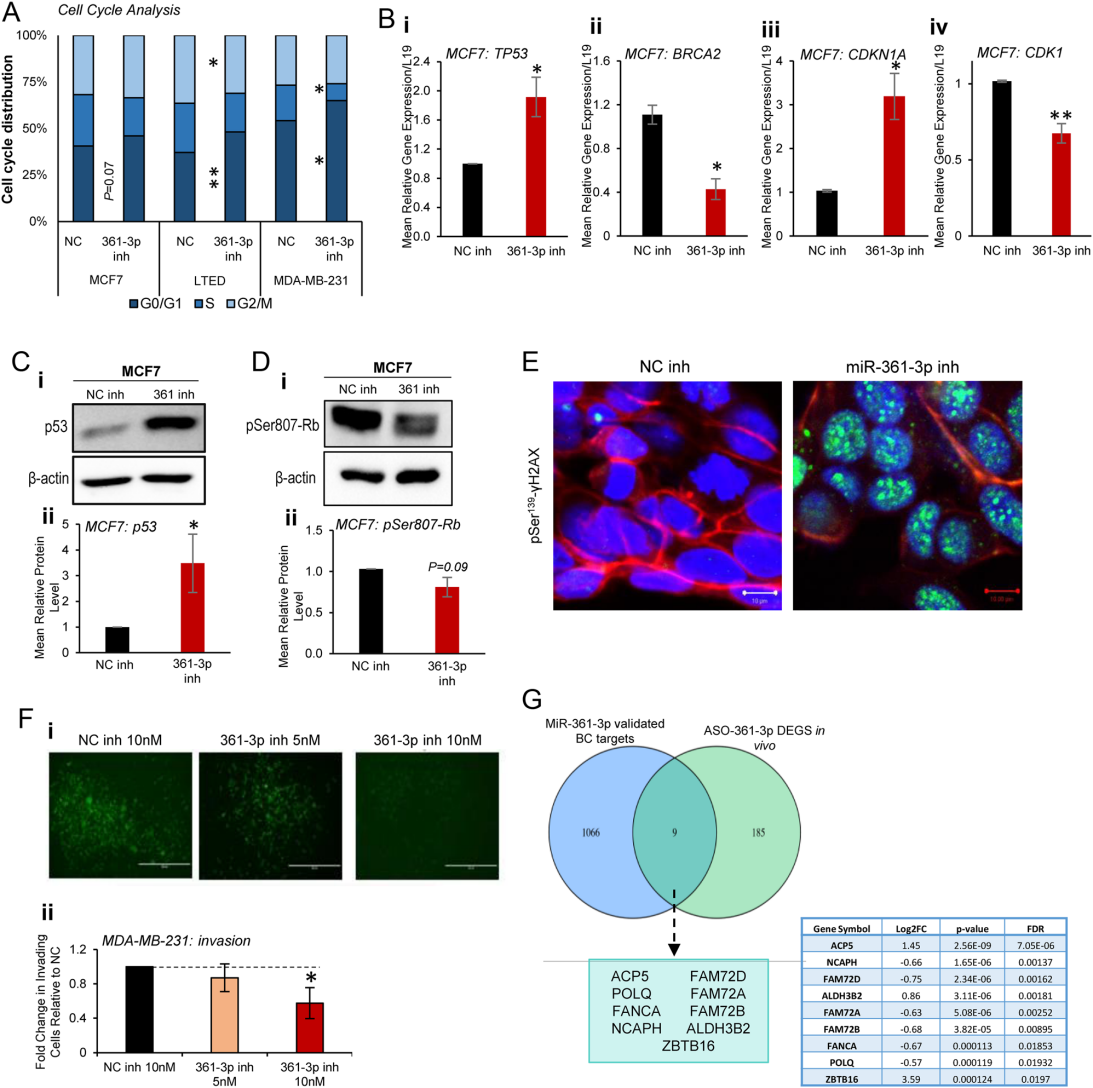
miR-361-3p inhibitor induces DNA damage and arrests breast cancer cells in G0/G1 phase of cell cycle. (A)Muse^TM^ analysis of cell cycle distribution of MCF7, LTED and MDA-MB-231 cells transfected with 20 nM miR-361-3p inhibitor or NC for 48 h. Columns: mean percentage of cells in each cell cycle phase for three independent experiments. Asterix denotes significance relative to NC. (B)qRT-PCR analysis of *TP53*, *BRCA2*, *CDKN1A* and *CDK1* transcript levels in MCF7 cells transfected with miR-361-3p or NC inhibitor (10 nM) for 48 h. Columns: mean fold change relative to *L19* and NC inhibitor ± s.e.m. for the three independent experiments performed in triplicate. (C,D)Western blot analysis of p53 and phospho-Ser^807^-Rb protein levels in MCF7 cells transfected with 20 nM miR-361-3p- or NC-inhibitor for 48 h. β-actin was used as a control for equal loading, and (ii) densitometry was performed using ImageJ. Columns:mean normalised protein levels ± s.e.m. relative to β-actin and NC for three independent experiments. (E)Immunofluorescent microscopy analysis of pSer^139^-γH2AX protein levels in MCF7 cells transfected with 10 nM NC or miR-361-3p inhibitor for 72 h. Green = pSer^139^-γH2AX, blue = nuclei and red = cytoskeleton. Scale bar = 10 µm. (F)24-h gelatin invasion assay analysis of MDA-MB-231 cells prior-transfected with miR-361-3p or NC inhibitor (5 or 10 nM) for 48 h. Representative fields are shown. Columns: mean fold change in the number of invading cells relative to NC inhibitor for three independent repeats ± s.e.m. (G)Venn diagram showing the overlap between significantly differentially expressed genes (DEGs) identified by RNA-seq of ASO-361-3p- vsASO-NC-treated MCF7 xenograft tumours and predicted/validated miR-361-3p targets (obtained from predictive (miRDB and TargetScan) and validated databases (AGO-PAR-CLIP and miRTarBase)). *P ≤ 0.05, **P ≤ 0.01.

To further delineate the mechanisms by which miR-361-3p inhibition represses *in vivo* BC tumour growth, RNA-seq analysis was performed on tumours excised from ASO-361-3p- and ASO-NC-treated mice. Top ASO-361-3p DEGs are shown in Supplementary Table 5 and Fig. 5A. Figure 5B shows a hierarchical clustering analysis of 194 genes identified as significantly differentially expressed between ASO-361-3p- and ASO-NC-treated tumours with FDR < 0.05. qRT-PCR confirmed decreased expression of top RNA-seq-identified DEGs *TGFB2*, *AMIGO2* and *ANKRD30A* and increased expression of *ZBTB16* in response to ASO-361-3p in MCF7 xenografts (Supplementary Fig. 15). GSEA revealed depletion of ASO-361-3p DEGs for transcripts involved in cell cycle-related progression (E2F targets, G2M checkpoints, mitotic spindle), immune response (IFNy and IFNa response), EMT processes and DNA repair (Fig. 5C). To confirm miR-361-3p inhibitor modulation of cell cycle progression, PI flow cytometry was performed. This revealed a significant increase in the percentage of MCF7, LTED and MDA-MB-231 cells in G0/G1, and a decrease in cells G2/M and S-phase, supporting cell cycle arrest at the G1/S transition, or entry into quiescence (G0) upon miR-361-3p inhibition (Fig. 6A). Notably, the top three GSEA-identified, ASO-361-3p-dysregulated pathways all contain *TP53* (upregulated) and *BRCA2* (downregulated) – both E2F target genes and regulators of cell cycle progression. qRT-PCR confirmed significant upregulation of *TP53* (1.9-fold) and downregulation of *BRCA2* (2.6-fold) in response to miR-361-3p inhibition, with concomitant increases in p53 protein levels (Fig. 6B and C, Supplementary Fig. 16). *CDKN1A* (p21) transcript levels were also significantly increased in response to miR-361-3p inhibition (3.2-fold – Fig. 6Biii), consistent with increases at the protein level observed by Western blotting (Fig. 2B). Since p21 functions as a critical regulator of G1/S transition (Georgakilas *et al.* 2017), its upregulation in response to miR-361-3p inhibition is consistent with the G1 arrest noted upon cell cycle profiling (Fig. 6A). p53-mediated induction of p21 represses various cyclin/CDK complexes, reducing their ability to phosphorylate and repress retinoblastoma protein (RB) through phosphorylation at Ser807/811 (Georgakilas *et al.* 2017). Reduced RB phosphorylation enhances its association with E2F transcription factors (Knudsen & Wang 1997), thus preventing the transcription of genes necessary for G1/S transition (Hiebert *et al.* 1992, Giacinti & Giordano 2006). We thus hypothesised that miR-361-3p inhibitor-mediated increase of p53 and p21 could result in hypophosphorylated RB at Ser807. Indeed, miR-361-3p inhibition reduced phospho-Rb (Ser807) protein levels (Fig. 6D, Supplementary Fig. 16), supporting the induction of cell cycle arrest at G1/S via p53 and p21.

P53 is activated in response to extrinsic or intrinsic stresses such as DNA damage or mitotic spindle in order to promote the repair of damaged DNA or induce apoptosis through modulation of target gene expression (Hafner *et al.* 2019). GSEA revealed depletion of miR-361-3p inhibitor-regulated genes for DNA repair transcripts, suggesting that miR-361-3p may induce DNA damage or inhibit its repair. To assess this, the formation of phospho-γH2AX (Ser^139^) nuclear foci (a marker of DNA damage) was assessed by immunofluorescence. The large number of foci observed in miR-361-3p inhibitor- vsNC inhibitor-transfected MCF7 cells (Fig. 6E, Supplementary Fig. 17) confirmed that miR-361-3p inhibitor promotes DNA damage or inhibits its repair.

A further GSEA-identified *in vivo* miR-361-3p inhibitor-downregulated pathway was epithelial-to-mesenchymal transition (EMT) – the cellular programme through which epithelial cells gain mesenchymal features, that aid their migration, invasion and metastasis to secondary sites (Thiery *et al.* 2009, Pastushenko *et al.* 2018). To assess the impact of miR-361-3p inhibition on this process, transwell gelatin invasion assays were performed in GFP-expressing MDA-MB-231 cells. miR-361-3p inhibitor significantly decreased BC cell invasion (Fig. 6F).

We next sought to identify miR-361-3p target genes most relevant to ASO-361-3p phenotype. The list of 194 DEGs likely comprises both direct and indirect miR-361-3p targets. Identification of potential targets directly regulated by miR-361-3p in BC tumours *in vivo* was achieved by integrating DEGs (*P* < 0.05) from xenograft RNA-seq analysis with 1075 predicted and/or validated miR-361-3p targets. Predicted targets were obtained from miRDB and TargetScan databases (Agarwal *et al.* 2015, Wong & Wang 2015), whilst validated targets were derived from MCF7 AGO-PAR-CLIP-seq data (Hamilton *et al.* 2016) (see Supplementary Table 6) and miRTarBase. From this, nine genes differentially expressed in response to ASO-361-3p *in vivo* were found to be direct miR-361-3p targets (Fig. 6G), including ACP5 (tartrate-resistant acid phosphatase), POLQ (DNA polymerase theta), NCAPH (Non-SMC Condensin I Complex Subunit H), ALDH3B2 (Aldehyde dehydrogenase family 3 member B), ZBTB16 (Zinc finger and BTB domain containing 16), FAM72 family and FANCA (Fanconi Anaemia Complementation Group A).

**Figure 7:**
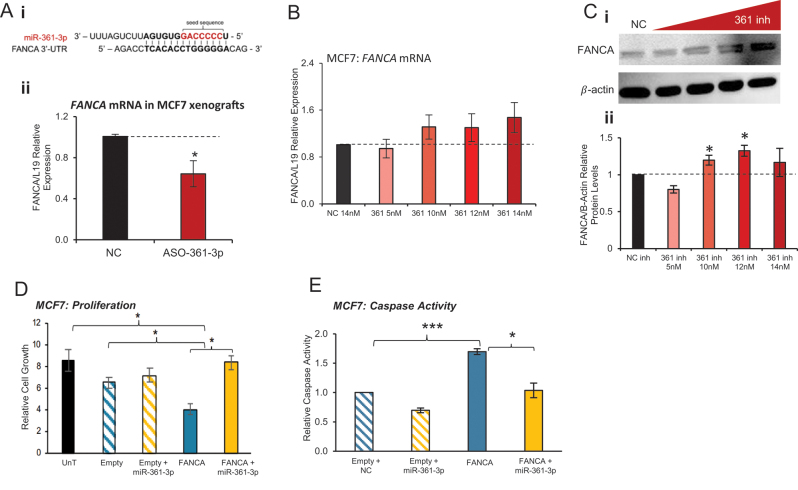
miR-361-3p effects are partially attributable to FANCA targeting in breast cancer. (A,B)qRT-PCR analysis of FANCA transcript levels in (A)MCF7 xenografts treated with ASO-361-3p vsASO-NC for 18 days and (B)MCF7 cells transfected with the indicated concentrations of miR-361-3p inhibitor for 6 days. Columns: mean fold change relative to L19 and NC ± s.e.m. (C)Western blot analysis of FANCA protein levels in MCF7 cells treated with 5, 10, 12 or 14 nM miR-361-3p inhibitor for 6 days. (ii) Densitometry was performed using ImageJ and protein levels are shown relative to β-actin for three independent experiments ± s.e.m. (D)SRB proliferation assay analysis of MCF7 cells transfected with FANCA ± miR-361-3p mimic (30 nM) for 3 days. Columns:mean cell growth relative to UnT cells for three independent experiments ± s.e.m. (E)Caspase 3/7-Glo assay analysis of MCF7 cells transfected with FANCA ± miR-361-3p mimic (30 nM) for 3 days. Columns:mean relative caspase activity relative to empty plasmid-transfected cells for three independent experiments ± s.e.m. *P ≤ 0.05, **P ≤ 0.01, ***P ≤ 0.0001.

The Fanconi anaemia proteins together with BRCA1/FANCS and BRCA2/FANCD1 act in a common pathway, FANCA/BRCA pathway, to coordinate the cellular response to DNA damage, driving DNA repair through homologous recombination (HR). Upon DNA damage, sensor proteins ATM and ATR trigger the formation of the FANCA protein complex, including A, C, E, F and G. Once formed, this complex ensues the monoubiquitylation of FANCD2. In turn, activated FANCD2 translocates to the DNA damage sites in the chromatin where the DNA-repair foci containing BRCA2/FANCD1 are located (García & Benítez 2008). Since FANCA was shown to be regulated by miR-361-3p inhibitor *in vivo*, and has been identified as a direct miR-361-3p target (Fig. 7Ai) and as BRCA2 transcript levels are reduced following miR-361-3p inhibitor transfection (Fig. 6Bii), we investigated the involvement of FANCA in miR-361-3p inhibitor phenotype. qRT-PCR validated reduced FANCA transcript levels in MCF7 xenografts following ASO-361-3p administration (Fig. 7Aii, Supplementary Fig. 18). Despite this, miR-361-3p inhibitor treatment *in vitro* increased FANCA mRNA levels in MCF7 cells in a dose-dependent manner (Fig. 7B). This inconsistency may be attributable to differences between long- and short-term miR-361-3p inhibitor effects, or miR-361-3p translational repression vstranscript degradation. Western blot analysis revealed dose-dependent significant increases in FANCA protein levels in response to miR-361-3p inhibitor (Fig. 7C), supportive of canonical miR-361-3p targeting of FANCA transcript.

Next, functional studies upon overexpression of FANCA levels were carried out in MCF7 cells. Compared to the control plasmid, FANCA overexpression significantly reduced MCF7 cell growth, which was rescued by miR-361-3p (Fig. 7D, Supplementary Fig. 18), confirming the phenotypic relevance of miR-361-3p targeting of FANCA and suggesting that effects of miR-361-3p inhibitor in cell growth are possibly mediated through FANCA increase. Similarly, FANCA overexpression significantly increased caspase activity in MCF7 compared to the control (control plasmid and NC mimic), which was abrogated by miR-361-3p mimic (Fig. 7E). This suggests that miR-361-3p inhibitor effects on apoptosis are possibly mediated at least partially by targeting FANCA.

**Figure 8:**
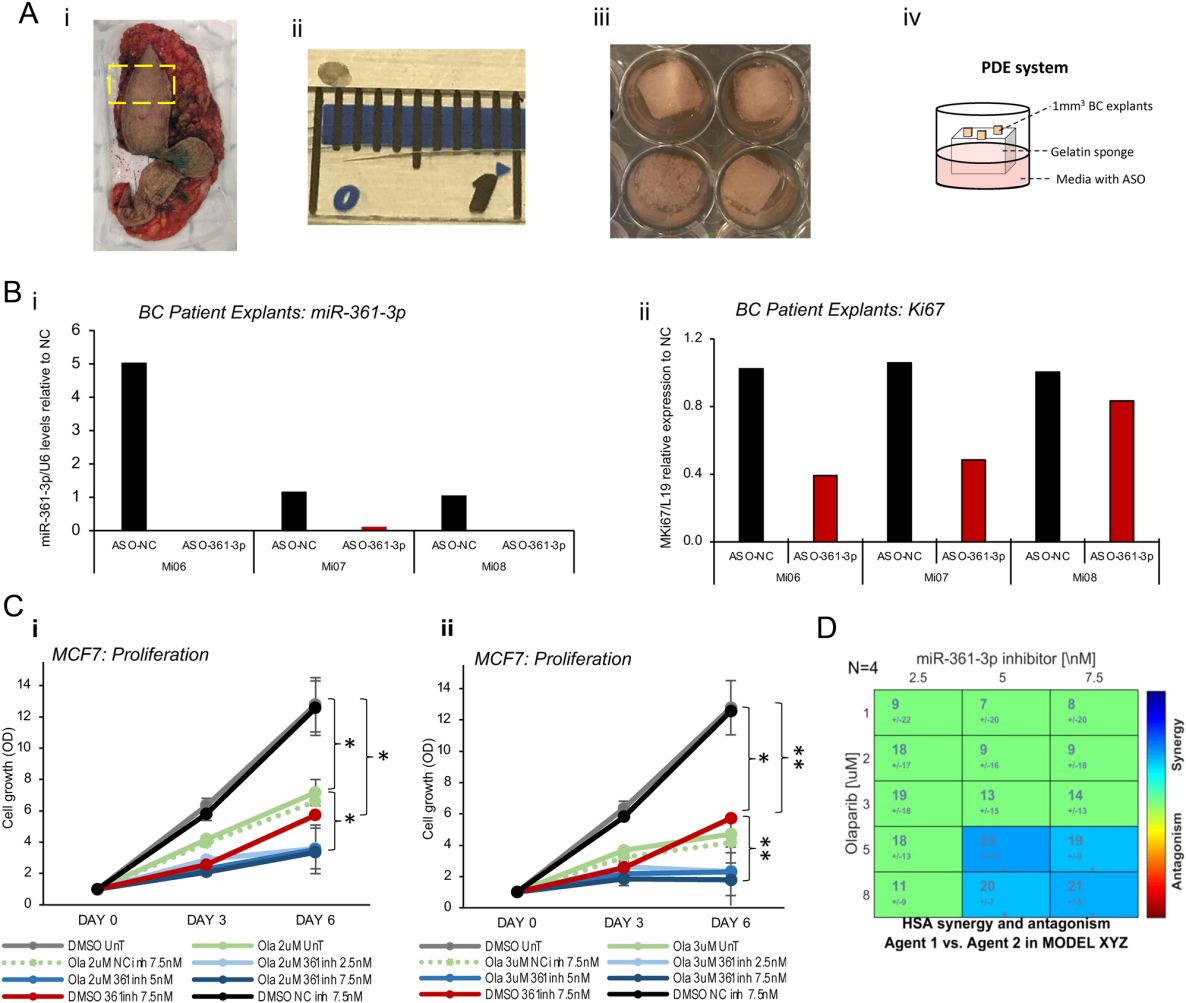
miR-361-3p inhibitor reduces breast cancer tumour explant proliferation *ex vivo* and synergises with olaparib *in vitro.* (A)Description of tumour explant culture method: i) biopsy gun or scalpel is used to remove tumour sample (tumour area is underneath the region outlined with yellow dashed line), ii)tumour tissue is dissected into 1 mm^3^ pieces, which are iii)cultured on a gelatin sponge soaked with media containing ASO-361-3p or ASO-NC, iv) schematic representation of the patient-derived explant (PDE) system. (B)qRT-PCR analysis of (i)miR-361-3p and (ii)Ki67 expression in BC patient explants treated with ASO-361-3p or ASO-NC for 48 h. U6 and L19 were used for normalisation, respectively. Columns: expression fold-change relative to ASO-NC treatment. (C)SRB assay analysis of MCF7 cell proliferation following transfection with negative control (NC) or miR-361-3p inhibitors (0–7.5 nM) alone or in combination with olaparib (2 µM – (i)*,* 3µM – (ii)) for 6 days. Points represent relative cell growth (mean absorbance per condition at day 3 or 6 relative to day 0 absorbance) ± s.e.m. for three independent experiments performed in quadruplicate. (D)Heatmap displaying the extent of synergy or antagonism between the miR-361-3p inhibitor and olaparib in MCF7 BC cells based on the highest single agent (HSA) model.

To demonstrate miR-361-3p therapeutic efficacy in a clinically relevant, near-patient model, three BC explants (Fig. 8A) were treated with ASO-361-3p for 48 h. Patient tumour clinical and pathological features are shown in Supplementary Table 7. ASO-361-3p was shown to dramatically reduce endogenous miR-361-3p levels in all explants (Fig. 8B), with a significant concomitant reduction in explant proliferation. PARP inhibitors (PARPi) are effective and in routine clinical use for advanced BC patients with BRCA1/BRCA2 gene mutations (Paluch-Shimon & Cardoso 2021). Given that miR-361-3p inhibition significantly reduced BRCA2 expression (Fig. 6Bii), we hypothesised that miR-361-3p inhibitor may sensitise BC cells to PARP inhibition. To this end, MCF7 cells were transfected with 0–7.5 nM miR-361-3p inhibitor ± Olaparib (2 and 3 µM). miR-361-3p inhibitor was shown to significantly enhance PARPi-mediated BC growth suppression (Fig. 8C) at both PARPi concentrations. To further investigate the effect of miR-361-3p inhibitor and olaparib co-treatment, Combenefit software was used to study the interaction between the treatments (Di Veroli *et al.* 2016). As shown in Fig. 8D, this identified a synergistic effect between miR-361-3p inhibitor (at 5 and 7.5 nM) and olaparib (at 5 and 8µM) using the highest single agent (HSA) model as reference (*n* = 4).

## Discussion

Cancer cells employ many mechanisms to circumvent the effects of drugs and promote resistance, including evasion of apoptosis. Since miRs play key roles in the regulation of tumourigenic processes, we hypothesised that miRs may alter apoptosis in drug-responsive and -resistant BC and could be exploited for therapeutic benefit, particularly since several miR ASOs are currently in clinical trials in cancer. High-throughput miR inhibitor screening identified miR-361-3p inhibitor as a potent inducer of apoptosis and repressor of proliferation in MCF7, TamR and LTED cells. Importantly, miR-361-3p mimic was able to rescue miR-361-3p inhibitor-repressed BC cell proliferation, confirming the specificity of miR-361-3p inhibitor effects. That miR-361-3p inhibitor significantly induced early-stage apoptosis in FulvR and MDA-MB-231 cells suggests that it can induce apoptosis across BC subtypes and in an ER-independent manner.

Antibody array and Western blot analyses revealed loss of apoptosis inhibitors, C-IAP and X-IAP, and increased p21 and p27 protein levels in response to miR-361-3p inhibition. X-IAP functions predominantly as a component of the intrinsic apoptosis pathway, whilst C-IAP can regulate both extrinsic and intrinsic pathways (Graber & Holcik 2011). This supports apoptotic plasticity in response to miR-361-3p inhibition, and that miR-361-3p inhibitor can activate both arms of the apoptotic cascade. Since extrinsic apoptosis activation requires binding of TRAIL, TNFα or FasL to cell surface receptors (Carneiro & El-Deiry 2020*a*), it would be of interest to determine whether miR-361-3p inhibitor can induce apoptosis in a paracrine manner, for example, through treatment of naïve cells with conditioned medium from miR-361-3p inhibitor-transfected cells.

Functional assays showed that p21 protein levels are also substantially increased following miR-361-3p inhibitor treatment, which is upregulated by p53 in response to DNA damage (Zlotorynski 2016). Since both DNA damage and p53 protein levels are also increased upon miR-361-3p inhibition, this may suggest that DNA damage-mediated p53 activation of p21 could indirectly induce apoptosis in response to miR-361-3p inhibition through repression of key cell cycle-promoting factors, CDK1 and CDK2, leading to apoptosis when repair of miR-361-3p-induced DNA damage fails. Indeed, CDK1 transcript levels are reduced by miR-361-3p inhibitor treatment (Fig. 6Biv). This is consistent with the block in cell cycle progression at G1/S observed following miR-361-3p inhibition.

Similarly to p21, miR-361-3p inhibitor-upregulated p27 (encoded by CDKN1B) may also induce apoptosis through regulation of cell cycle progression: p27 protein blocks activity of CDK2–cyclin E and CDK4–cyclin D complexes at G1/S boundary, and within G-phase, respectively (again consistent with miR-361-3p inhibitor cell cycle analysis data). Importantly, the effects of miR-361-3p inhibition on apoptosis are in agreement with its ability to robustly repress proliferation across endocrine-responsive (MCF7) and endocrine-resistant (TamR and LTED) cells. These findings are also consistent with increased miR-361-3p expression across all BC subtypes in the METABRIC data set, suggestive of potential oncogenic activity. This is further supported by the trend toward reduced survival of BC patients with high miR-361-3p tumour levels.

Inhibition of miR-361-3p shows considerable potential as a BC therapeutic, as systemic administration of ASO-361-3p significantly repressed breast tumour growth *in vivo,* with no evidence of overt toxicity. Importantly, our data show efficient target regulation: miR-361-3p levels are reduced by over 90% in all ASO-361-3p-administered tumours. However, ASO-361-3p was also able to significantly reduce miR-361-3p levels in the spleen and lungs of treated mice. This may support investigations into the targeted delivery of ASO-361-3p. Notably, whilst ASO-361-3p significantly reduced xenograft cell proliferation (as evidenced by reduced MCM4 protein levels), no alterations in cleaved PARP were observed. This suggests that the mechanism-of-action of miR-361-3p inhibitor across short-term *in vitro* experiments (apoptotic induction) may shift during longer term *in vivo* experiments to favour proliferative repression over apoptotic induction, perhaps as a result of altered p53 expression.

In order to fully exploit miR-361-3p inhibition, it is vital to fully comprehend its gene regulatory activities. We conducted RNA-seq analyses of ASO-miR-361-3p-treated vsASO-NC-treated MCF7 xenografts. Pathway analysis showed miR-361-3p is involved in cell cycle (E2F targets, G2M checkpoints, mitotic spindle), DNA repair, EMT processes and immune response (IFNy and IFNa response). These are consistent with the miR-361-3p inhibitor-induced G1/S block observed by flow cytometry, growth suppression *in vivo* and DNA damage *in vitro*. A potential role for miR-361-3p in suppressing EMT is supported by the decreased invasion of MDA-MB-231 cells following miR-361-3p inhibitor transfection. The contribution of cell cycle process to miR-361-3p inhibitor phenotype is underscored by the increase in protein levels of the cell cycle master regulator, p53, in response to miR-361-3p inhibitor, and accompanying reduction of Ser^807^-phosphorylated Rb, since p21 (CDKN1A) repression of cyclin/CDK complexes reduces their ability to phosphorylate and repress Rb, leading to increased Rb inhibition of E2F transcription factors driving G1/S cell cycle transition (Hiebert *et al.* 1992, Knudsen & Wang 1997, Giacinti & Giordano 2006).

Integration of ASO-361-3p DEGs in BC xenografts with validated and predicted miR-361-3p targets identified FANCA as a direct miR-361-3p target, which alongside other FANC family members and BRCA proteins, coordinates cellular response to DNA damage, driving DNA repair through HR (García & Benítez 2008). FANCA transcript levels were decreased by 18d ASO-361-3p treatment *in vivo* but increased by 6d miR-361-3p inhibitor treatment at both transcript and protein levels *in vitro*. An increase in FANCA protein levels in response to miR-361-3p inhibition is consistent with canonical miR-361-3p targeting of this gene. Indeed, FANCA 3’UTR displays perfect miR-361-3p seed complementarity across 14nt. Discrepancies between *in vitro* and *in vivo* FANCA modulation may be attributable to shifting mechanism-of-action of miR-361-3p inhibition between short-term and long-term experiments (as discussed earlier), or potentially miR-361-3p repression of FANCA translation, rather than transcript degradation. Importantly, FANCA overexpression phenocopied effects of miR-361-3p inhibition on MCF7 proliferation and apoptosis (Fig. 7D and E), and miR-361-3p mimic rescued FANCA-mediated growth suppression and apoptotic induction, suggesting that miR-361-3p effects are mediated, at least in part, through FANCA targeting.

It is notable in the context of miR-361-3p inhibitor upregulation of FANCA that the same treatment reduced BRCA2 transcript levels. Combined with the observed DNA damage upon miR-361-3p inhibitor treatment, this may suggest that miR-361-3p inhibitor-induced BRCA2 loss results in reduced efficacy of HR. This would be hypothesised to sensitise BC cells to PARP inhibition, which blocks ssDNA break repair through PARP trapping (Paluch-Shimon & Cardoso 2021). Indeed, miR-361-3p inhibitor synergised with PARPi, supporting the involvement of BRCA2 in miR-361-3p inhibitor phenotype, and potentially supporting the clinical use of miR-361-3p inhibition as a PARPi-sensitising agent. Notably, POLθ (a low fidelity DNA repair enzyme with an important role in microhomology-end joining (Yousefzadeh *et al.* 2014)) was also identified as a direct miR-361-3p target. Since this shows synthetic lethality in BRCA1-deficient tumours (Ceccaldi *et al.* 2015, Mateos-Gomez *et al.* 2015), this supports further studies to confirm miR-361-3p inhibitor downregulation of POLθ, and whether it can sensitise BC cells to Polθ inhibition in BRCA-proficient vs-deficient tumours.

Finally, to provide proof-of-principle for the clinical use of miR-361-3p inhibitor in BC, three explants were treated with ASO-361-3p for 48 h. As well as significantly reducing endogenous target miR-361-3p levels in these tumours, ASO-361-3p also significantly reduced levels of proliferation marker, Ki67. This supports further preclinical studies into the use of miR-361-3p inhibitor as a novel BC therapeutic, and the identification of the subset of patients most likely to respond.

Little is known of the role of miR-361-3p in BC; however, it is generally reported that miR-361-3p has oncogenic activity across a number of malignancies. In prostate cancer, it was shown to stabilise the androgen receptor (AR) transcript, leading to increased AR activity that promotes proliferation, EMT, migration and invasion (Fletcher *et al.* 2019). miR-361-3p oncogenic activity was also partially attributable to its targeting of ARHGDIA and TAGLN2, and its levels were elevated in response to anti-androgen treatment of advanced PC patient-derived xenografts. In colorectal cancer (CRC), miR-361-3p is present within vesicles released by hypoxic CRC cells and can be transferred to normoxic CRC cells to repress apoptosis and accelerate proliferation (Li *et al.* 2021), consistent with the results in this study. miR-361-3p actions in different cell contexts will ultimately be driven by miR-361-3p levels and expression profiles of its target transcripts.

In conclusion, we have shown that miR-361-3p inhibition induces apoptosis and robust growth suppression *in vitro,in vivo* and *ex vivo* in drug-responsive and -resistant BC through activation of cell cycle checkpoints and potential induction of DNA damage. miR-361-3p is increased across all BC subtypes compared to normal tissue, and our studies provide proof-of-principle for pre-clinical exploration of miR-361-3p inhibitor as a new therapeutic option for the treatment of BC.

## Supplementary Material

Supplementary Material

Supplementary Data

## Declaration of interests

The authors have no conflicts of interest to declare.

## Funding

This work was supported by Rosetrees Trust http://dx.doi.org/10.13039/501100000833 Research Grants M192-F3 and M192-F4, an Imperial College London http://dx.doi.org/10.13039/501100000761 Research Fellowship (Fletcher), and a Leukemia and Lymphoma Society http://dx.doi.org/10.13039/100005189 Fellow Award (Hamilton). The authors gratefully acknowledge infrastructure support from Imperial Experimental Cancer Medicine Centre http://dx.doi.org/10.13039/100016338, Cancer Research UK http://dx.doi.org/10.13039/501100000289 Imperial Centre and National Institute for Health Research http://dx.doi.org/10.13039/100005622 (NIHR) Imperial Biomedical Research Centre (BRC).
