# Supplementary material for: Apoptosis-modulatory miR-361-3p as a novel treatment target in endocrine-responsive and endocrine-resistant breast cancer
Source: J Endocrinol. 2023 Feb 11;256(3):e220229. doi: 10.1530/JOE-22-0229 (PMC9986394; doi:10.1530/JOE-22-0229)
Supplement: Supplementary Material [file supplementary_material.pdf]

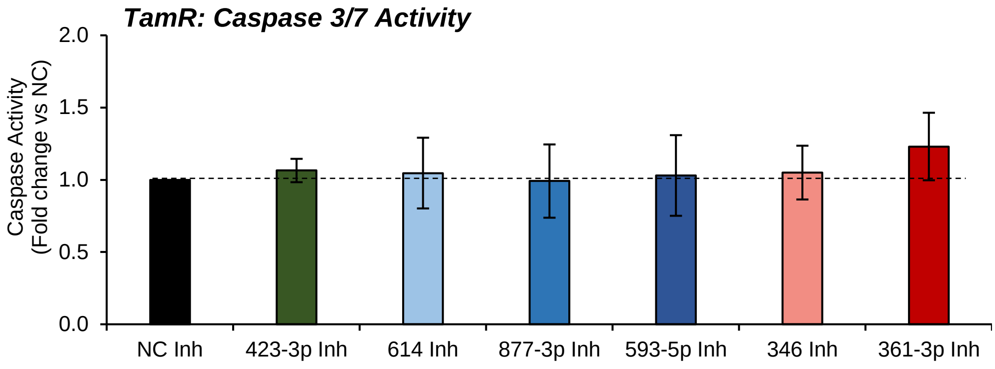

Figure S1: Inhibitors of miR-423-3p, -614, -877-3p, -593-5p, -346 and -361-3p do not significantly alter apoptosis in Tamoxifen-resistant MCF7/TaMR cells. Graphs show Caspase-3/7 Glo luciferase assay analysis of caspase-3/7 mediated apoptosis in TamR cells following transfection with miR-346, -361-3p, -423-3p, -614, -593-5p and -877-3p inhibitors (20nM) for 72h, normalised to cell number by sulforhodamine B (SRB) assay. Inhibitors are denoted as ‘Inh’. Columns: mean fold change ± SEM in caspase 3/7 activity at day three relative to non-targeting negative inhibitor control for three independent experiments performed in triplicate.

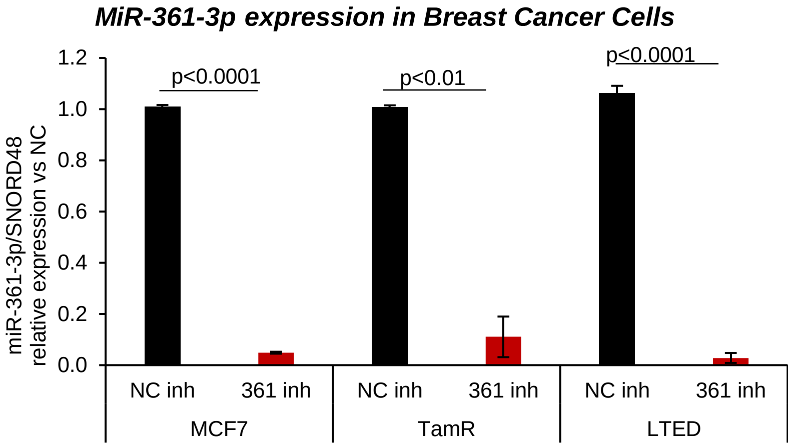

Figure S2: MiR-361-3p inhibitor reduces endogenous miR-361-3p levels in MCF7, TamR and LTED breast cancer cells. qRT-PCR analysis of endogenous miR-361-3p levels in MCF7, TamR and LTED cell following transfection with miR-361-3p inhibitor for 72h. Snord48 was used as normalisation gene. Columns: mean fold change normalised to Snord48 and shown relative to MCF7 NC inhibitor ± SEM for three independent experiments performed in triplicate. *P* values as indicated.

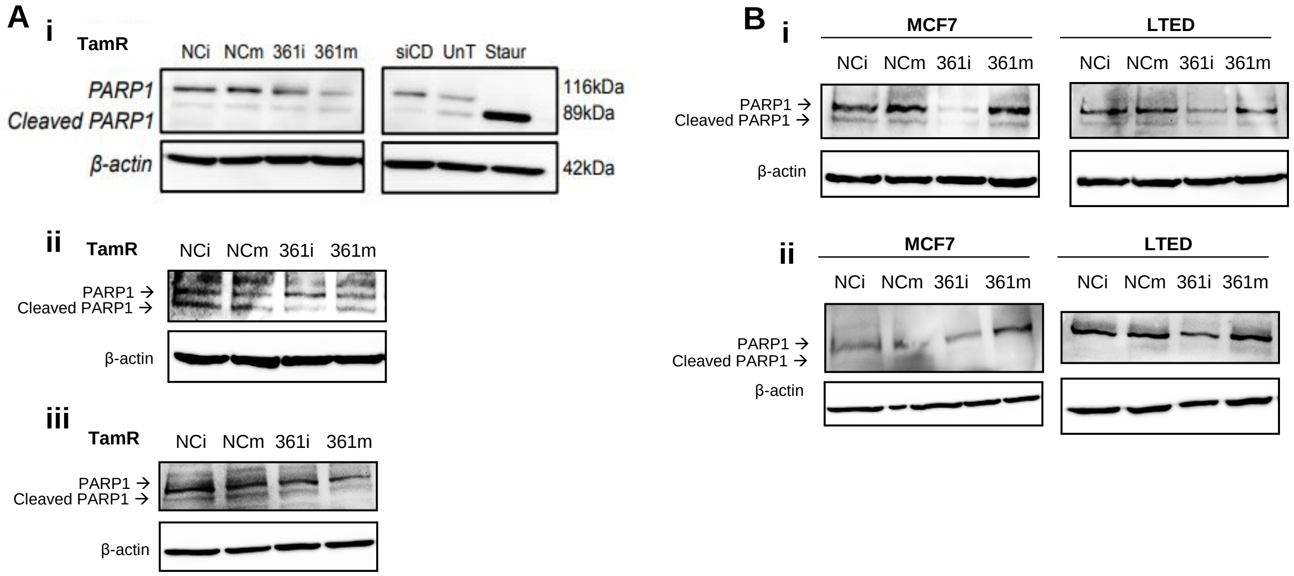

Figure S3. Assessing PARP Cleavage in TamR, MCF7 and LTED Cells in Response to MiR Inhibition. Western blot analysis of full-length and cleaved PARP1 protein levels in *A)* TamR and *B)* MCF7 and LTED cells transfected with miR-361-3p inhibitor (20nM), -mimic (30nM) or controls for 72h. β-actin was used as a loading control. Inhibitors are denoted as ‘i’, and mimics as ‘m’, respectively. Controls include negative control (NC) inhibitor and mimic-transfected cells, and siCD and staurosporine (‘Staur’) positive controls. Staurosporine treatment was performed 16h before cell harvesting. Biological replicates relating to **Fig 1E** are shown.

A

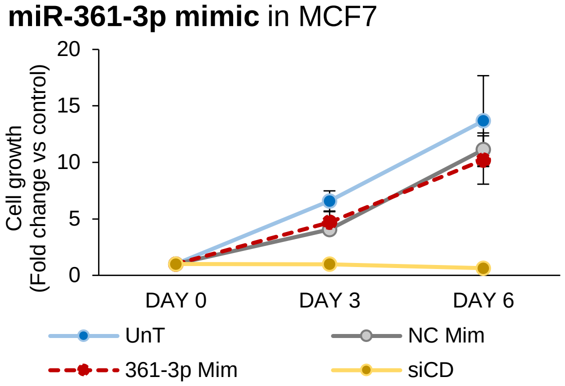

B

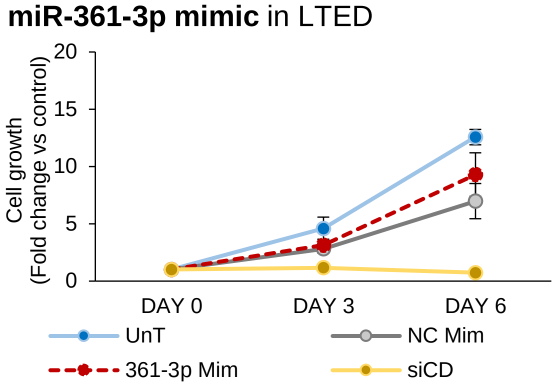

C

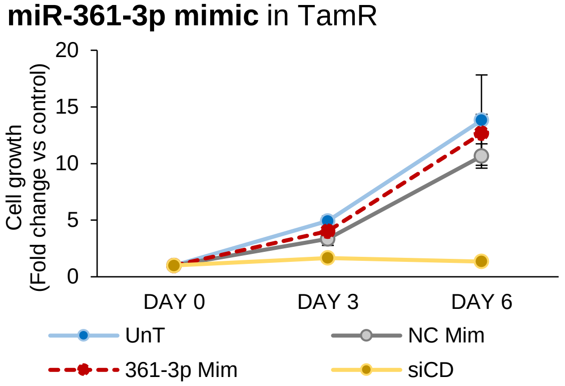

Figure S4: MiR-361-3p Mimic does not Alter Proliferation of MCF7, LTED or TamR Breast Cancer Cells. Sulphorhodamine B (SRB) assay analysis of MCF7 *(A)* and LTED *(B)* and TamR *(C)* proliferation following transfection with negative control (NC) and miR-361-3p mimics (30nM) or siCellDeath (siCD – 20nM) for 0-6 days. Points represent mean relative cell growth (mean absorbance per condition at day 3 or 6 relative to day 0 absorbance) ± SEM. siCD targets essential viability transcripts and is a positive control for growth inhibition. Mimics are denoted as ‘Mim’. Mock transfection is denoted as ‘UnT’.

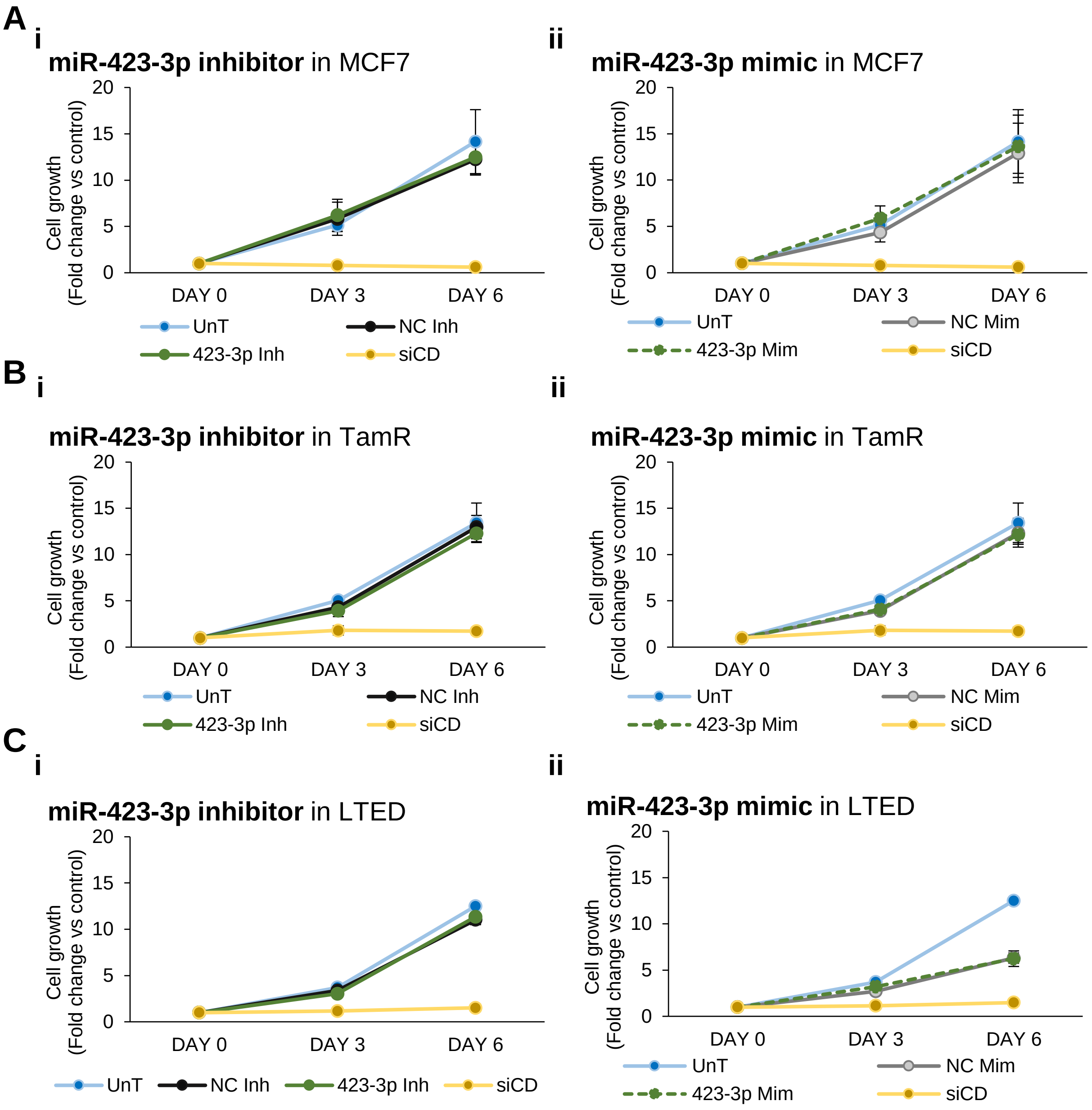

Figure S5: MiR-423-3p Modulation does not Alter Proliferation of MCF7, LTED or TamR Breast Cancer Cells. Sulphorhodamine B (SRB) assay analysis of MCF7 *(A)* and TamR *(B)* and LTED *(C)* proliferation following transfection with negative controls (NC) and miR-423-3p inhibitors (20nM) *(i)* or miR-423-3p mimics (20nM) *(ii)* for 0-6 days. Points represent mean relative cell growth (mean absorbance per condition at day 3 or 6 relative to day 0 absorbance) ± SEM. siCD targets essential viability transcripts and is a positive control for growth inhibition (20nM). Inhibitors are denoted as ‘Inh’, and mimics as ‘Mim’, respectively. Mock transfection is denoted as ‘UnT’.

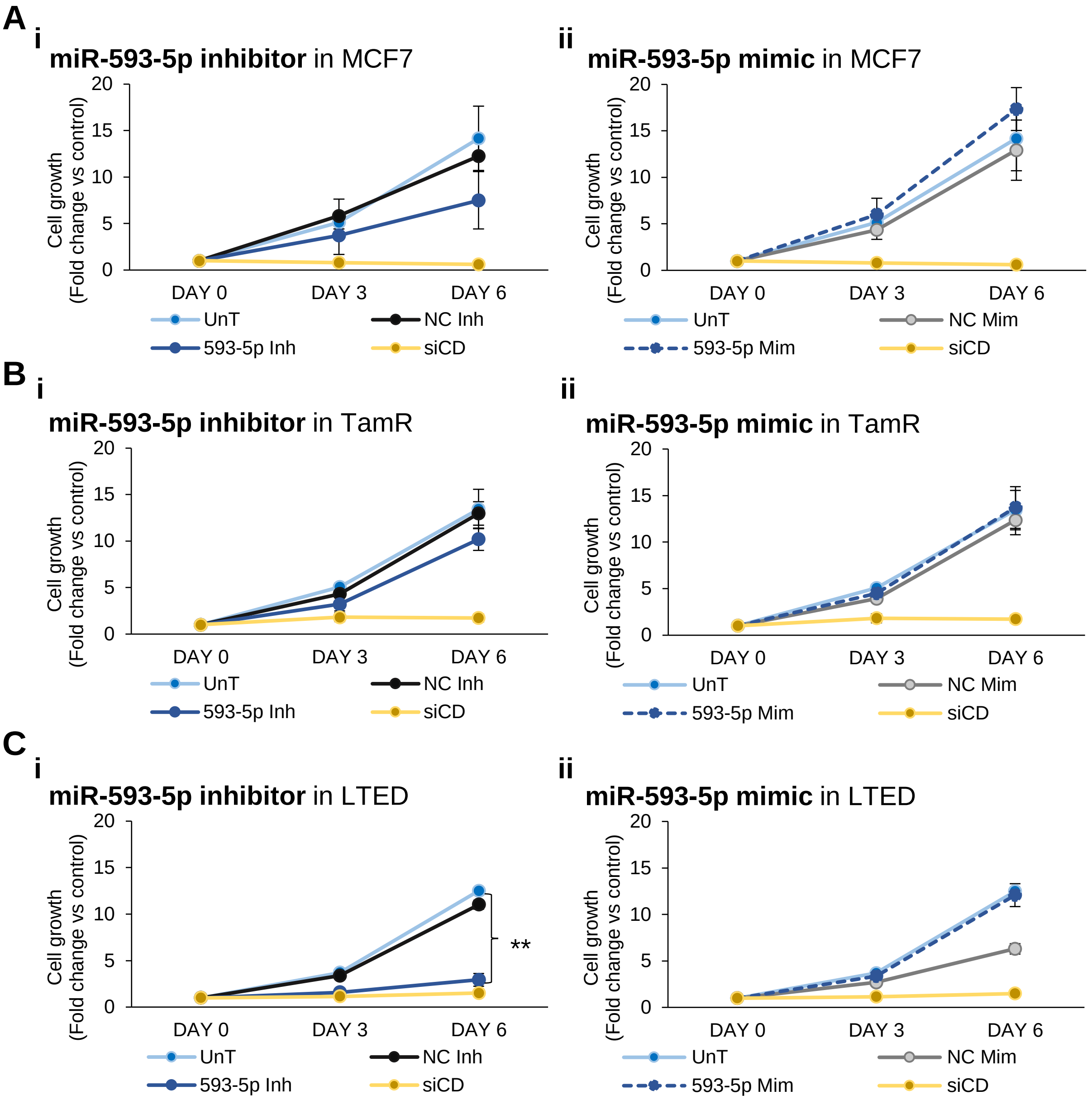

Figure S6: MiR-593-3p Inhibition Alters Proliferation of LTED Breast Cancer Cells. Sulphorhodamine B (SRB) assay analysis of MCF7 *(A)* and TamR *(B)* and LTED *(C)* proliferation following transfection with negative controls (NC) and miR-593-5p inhibitors (20nM) *(i)* or miR-593-5p mimics (20nM) *(ii)* for 0-6 days. Points represent mean relative cell growth (mean absorbance per condition at day 3 or 6 relative to day 0 absorbance) ± SEM. siCD targets essential viability transcripts and is a positive control for growth inhibition (20nM). Inhibitors are denoted as 'Inh', and mimics as 'Mim', respectively. Mock transfection is denoted as 'UnT'. (** P≤0.005).

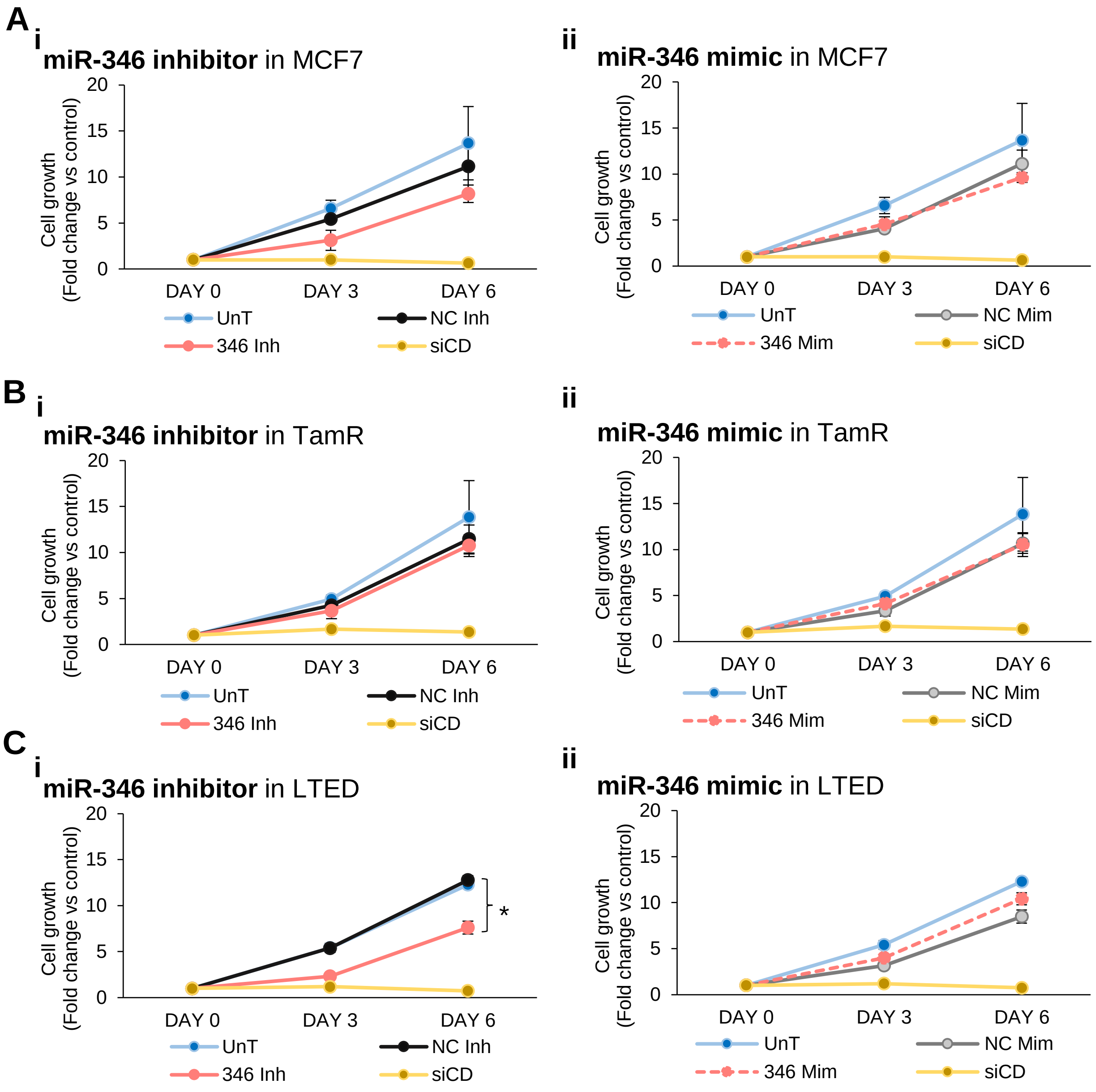

Figure S7: MiR-346 Inhibition Reduces Proliferation of LTED Breast Cancer Cells. Sulphorhodamine B (SRB) assay analysis of MCF7 *(A)* and TamR *(B)* and LTED *(C)* proliferation following transfection with negative controls (NC) and miR-346 inhibitors (20nM) *(i)* or miR-346 mimics (20nM) *(ii)* for 0-6 days. Points represent mean relative cell growth (mean absorbance per condition at day 3 or 6 relative to day 0 absorbance) ± SEM. siCD targets essential viability transcripts and is a positive control for growth inhibition (20nM). Inhibitors are denoted as 'Inh', and mimics as 'Mim', respectively. Mock transfection is denoted as 'UnT'. (* $P \leq 0.05$).

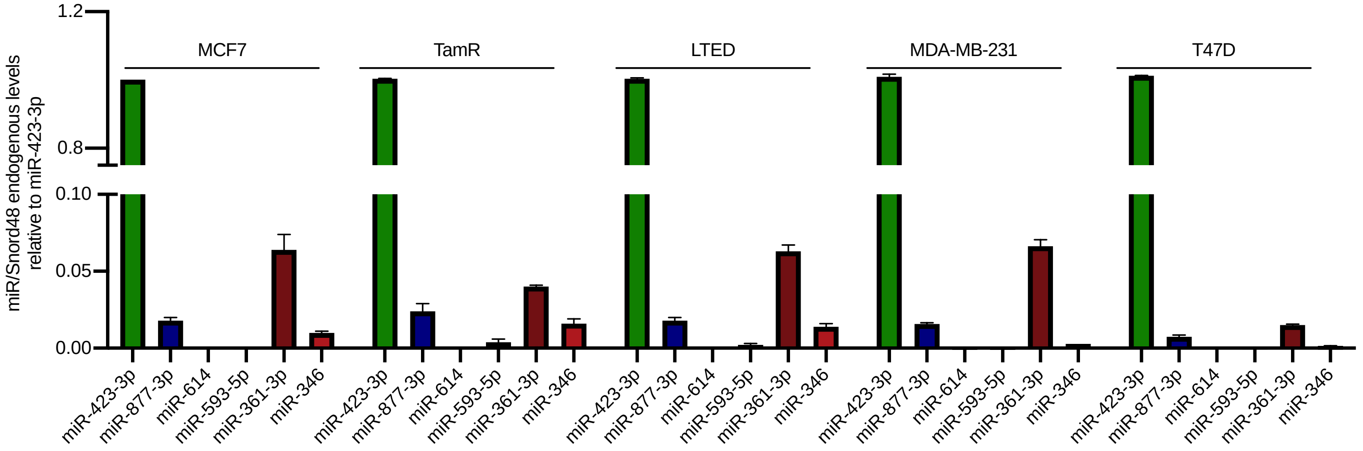

Figure S8. Endogenous levels of the panel of six miRs identified in by HTS in breast cancer cell lines. Graph shows qPCR analysis of endogenous levels of miR-423-3p, miR-877-3p, miR-614, miR-593-5p, miR-361-3p and miR-346 in MCF7, TamR, LTED, MDA-MB-231 and T47D cells. Snord48 was used as housekeeping normalisation gene, and fold change was calculated relative to respective miR-423-3p levels per cell line. Columns: mean fold change relative to Snord48 and miR-423-3p levels ± SEM for three independent experiments performed in triplicates.

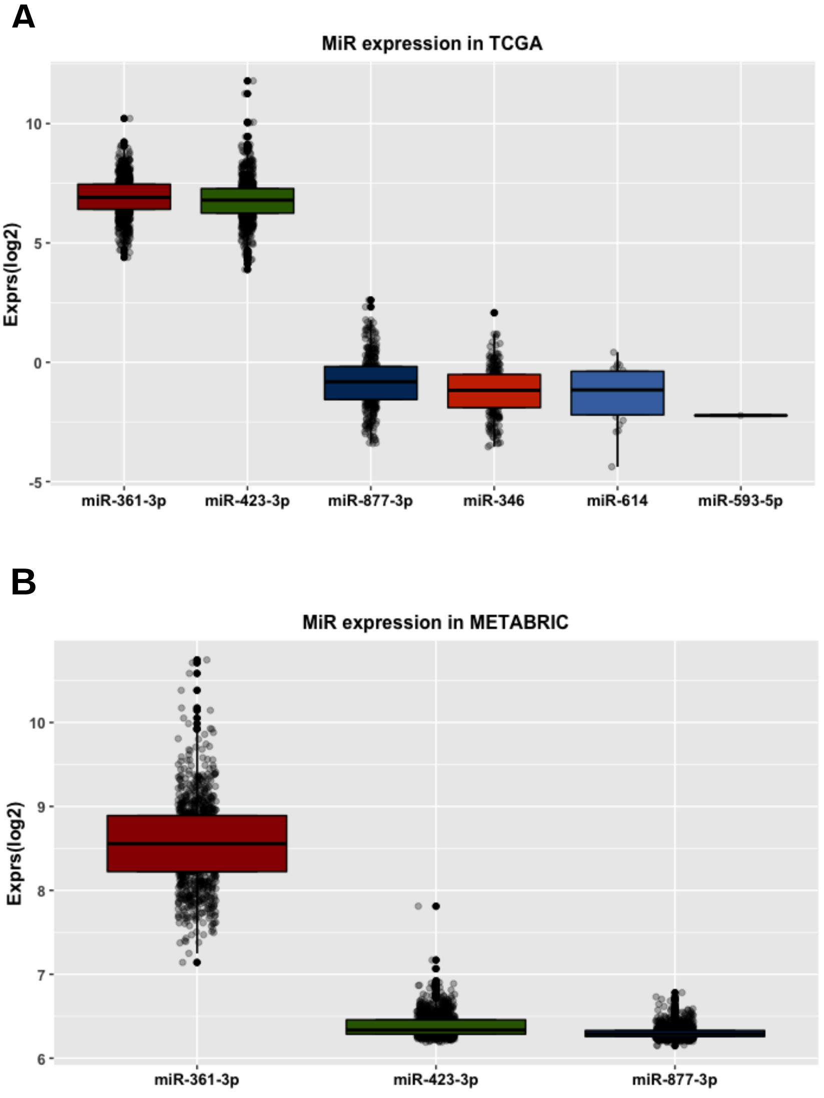

Figure S9. MiR-361-3p is the most expressed miR of the panel of six miRs from HTS selected for validation in the TCGA and METABRIC breast cancer datasets. *A) E*xpression levels (log2TPM) of the six miRs identified in the high-throughput screening in the TCGA dataset. A total of 764 women with invasive ductal carcinoma were analysed. This showed that miR-361-3p and -423-3p were the most highly expressed miRs in the panel. Following in expression were miR-877-3p, -346 and - 614, only detected in 343, 234 and 22 women, respectively. MiR-593-5p was the lowest expressed miR in the panel, with levels only detected in one sample. *B)* As for A, miR expression analysis of 985 patients with invasive ductal carcinoma in the METABRIC dataset. MiR-361-3p was the most expressed miR in the panel of six, followed by miR-423-3p and miR-877-3p. MiR-346, -614 and -593-5p were not detected in any of the 985 primary tumour samples.

mmu-miR-361-3p 5’ U<u>CCCCCAGGUGUGAUUC</u>UGAUUUGU 3’
hsa-miR-361-3p 5’ U<u>CCCCCAGGUGUGAUUC</u>UGAUUU 3’
ASO-miR-361-3p *3’ GGGGGTCCACACTAAG 5’*

Figure S10: ASO-361-3p targets hsa-miR-361-3p and mmu-miR-361-3p. ASO-361-3p is a 16nt long oligonucleotide (Qiagen) complimentary to both human (23nt) and mouse (25nt) miR-361-3p sequences, which differ in two bases (indicated in purple) at the 3’ end.

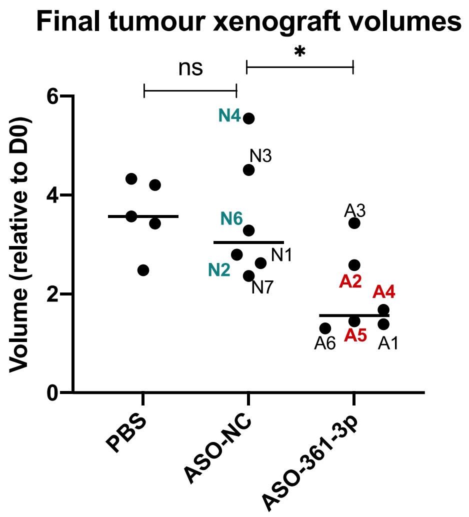

Figure S11: Final MCF7 tumour xenograft volumes following ASO-361-3p treatment for 18d. MCF7 xenografts were established on flanks of BALB/c nude mice, which were then randomly distributed into three treatment groups: 10mg/kg ASO-361-3p, ASO-NC or vehicle (PBS) for 18 days. The graph shows the median and individual final tumour volumes relative to day 0 tumour volume. Mice treated with ASO-361-3p are denoted with ‘A’ and the mouse number (1-6), and the ASO-NC treated mice are denoted with ‘N’ and the mouse number. Tumours used for RNA-sequencing are highlighted in green and red for ASO-NC and ASO361-3p treated mice, respectively (T-test, * p<0.05).

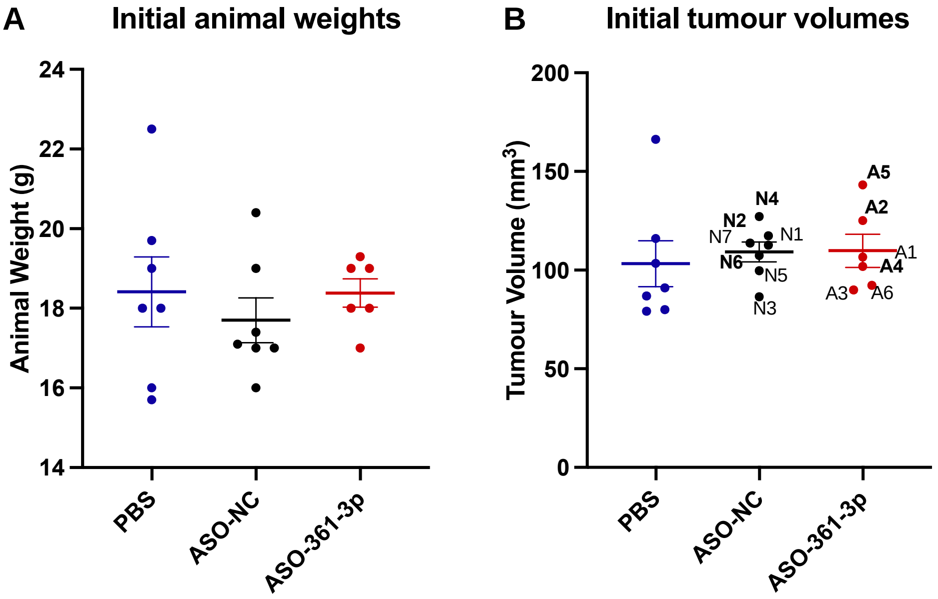

Figure S12: Initial animal weights and MCF7 tumour xenograft volumes were not different between treatment groups. MCF7 xenografts were established as described above and pre-treatment animal weights and tumour volumes were assessed. Animal numbers are labelled with a ‘P’, ‘N’ or ‘A’ and a number 1-7, for the PBS (denoted in blue), ASO-NC (denoted in black) or ASO-361-3p (denoted in red) treatment group, respectively.

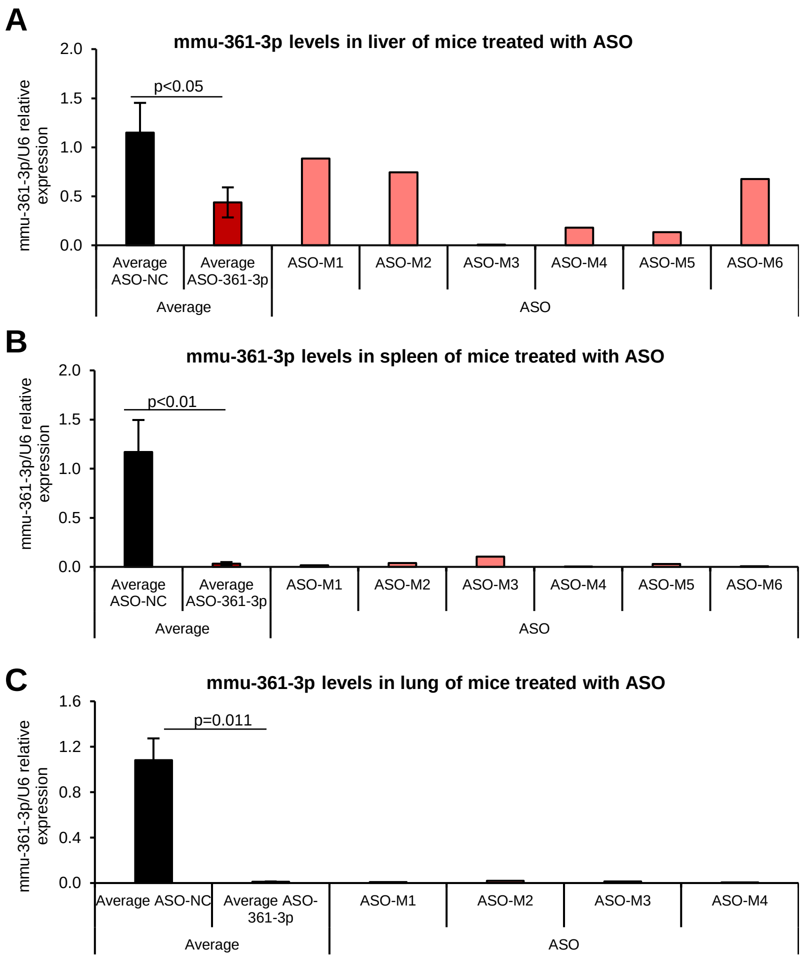

Figure S13: Treatment with ASO-361-3p significantly reduces mmu-miR-361-3p endogenous levels in mouse spleen and lung. BALB/c nude mice bearing MCF7 tumour xenografts were treated systemically twice a week for 18 days with ASO-361-3p, ASO-NC or vehicle (PBS) treatment. Graphs show qPCR analysis of endogenous levels of miR-361-3p in the liver *(A),* spleen *(B)* and lung (*C)* of mice following ASO-361-3p and ASO-NC 18-days treatment. U6 was used as normalisation gene. On the left, columns show the mean fold change relative to U6 and the average of ASO-NC ± SEM for the six and four independent experiments performed in triplicates. On the right, are the individual levels in each mice treated with ASO-361-3p.

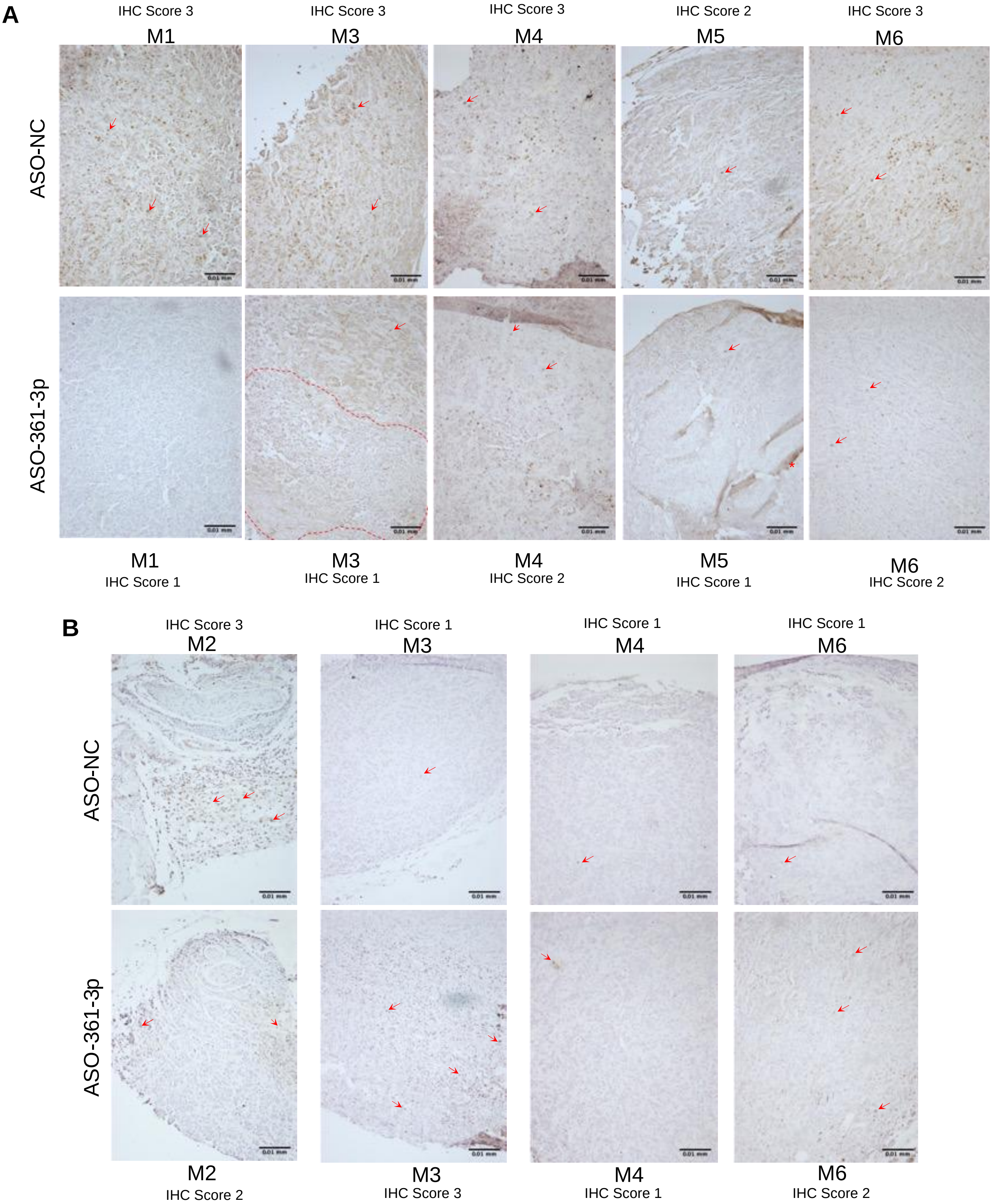

Figure S14: ASO-361-3p reduces tumour proliferation in MCF7 breast cancer xenograft tumours. Immunohistochemical analysis of *A)* MCM2 (proliferation marker) and *B)* cleaved PARP (apoptosis marker) protein levels in sections of formalin-fixed, paraffin-embedded MCF7 breast cancer xenografts from BALB/c nude mice treated systemically twice a week for 18 days with 10mg/kg ASO-361-3p, ASO-NC or vehicle (PBS). Results for 5 mice and 4 mice are shown for MCM2 and cleaved PARP, respectively. Brown colour denotes positive staining. Regions outlined in red refer to mouse stroma. Scale bar = 0.01mm. Assessment of IHC scores was made as reported for other protein immunohistochemical studies (Garcia De La Torre et al., 2006; Jin et al., 2001) with modifications. Scores were assigned according to the staining intensity and proportion of positive cells (examples of positive cells highlighted with red arrows), ranging from 1 to 3: IHC Score 1 corresponds to weak intensity in <10% cells, IHC Score 2 is a weak staining in ≤20% of cells or moderate staining in ≤10%; and IHC Score 3 is assigned to sections with moderate or strong staining in >10% cells.

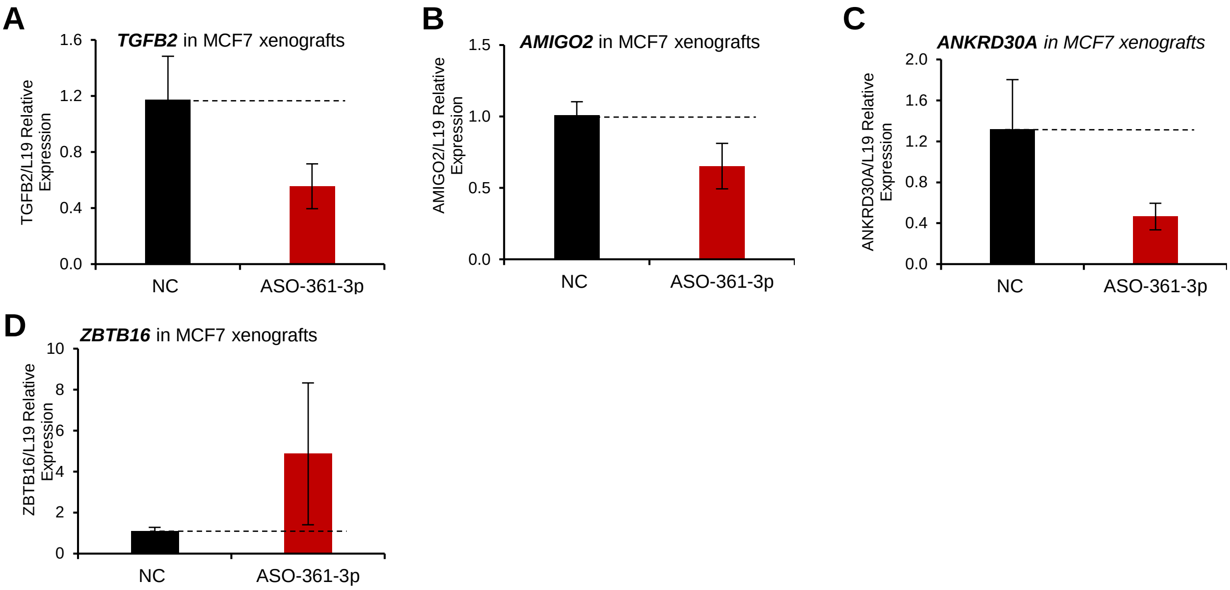

Figure S15. TGFB2, AMIGO2 and ANKRD30A Transcript Levels are Reduced in ASO-361-3p-treated MCF7 Xenografts. qRT-PCR analysis of *A) TGFB2*, *B) AMIGO2*, *C) ANKRD30A*, and *D) ZBTB16* transcript levels in MCF7 breast cancer xenograft tumours from Balb/c nude mice treated with 10mg/kg ASO-361-3p or ASO-NC for 18d (n=6/group). L19 was used as a normalization gene. *Columns:* mean transcript levels relative to L19 and NC treatment for six mice in technical triplicate.

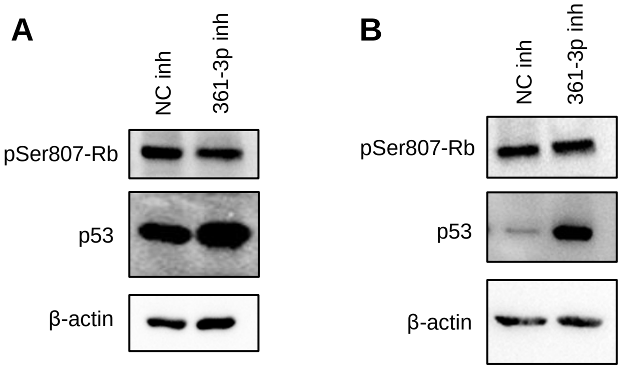

Figure S16. MiR-361-3p Inhibition Alters p53 and Phospho-Ser^{807}-Rb Protein Levels in MCF7 Breast Cancer Cells. *A, B)* Western blot analysis of p53 and phospho-Ser^{807}-Rb protein levels in MCF7 cells transfected with 20nM miR-361-3p- or NC-inhibitor for 48h. β-actin was used as a control for equal loading, and densitometry was performed using ImageJ. Biological replicates relating to **Fig 6C, D** are shown.

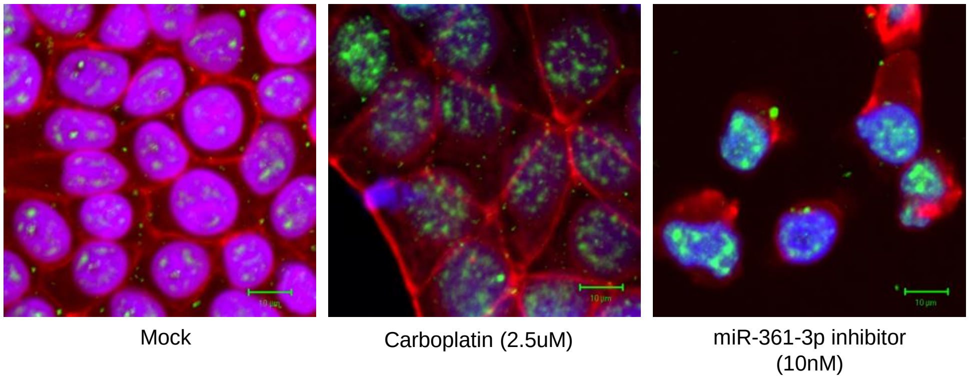

Figure S17. MiR-361-3p Inhibition Induces DNA Damage in MCF7 Breast Cancer Cells. Immunofluorescent microscopy analysis of pSer^{139}-γH2AX protein levels in MCF7 cells transfected with 10nM NC or miR-361-3p inhibitor for 72h. Green = pSer^{139}-γH2AX, blue = nuclei, red = cytoskeleton. Scale bar = 10µm. Biological replicates relating to **Fig 6E** are shown.

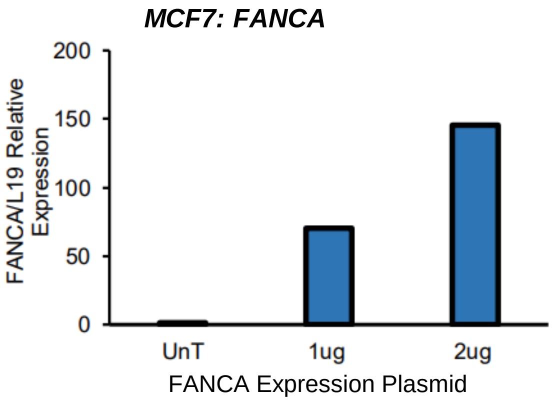

Figure S18. *FANCA* expression following plasmid transfection. qPCR analysis of *FANCA* transcript levels in MCF7 cells following transfection with indicated quantities (x-axis) of FANCA expression plasmid for 72h. L19 was used as a normalisation gene.
