# Supplementary material for: Apoptosis-modulatory miR-361-3p as a novel treatment target in endocrine-responsive and endocrine-resistant breast cancer
Source: J Endocrinol. 2023 Feb 11;256(3):e220229. doi: 10.1530/JOE-22-0229 (PMC9986394; doi:10.1530/JOE-22-0229)
Supplement: Supplementary Data [file supplementary_table_1.pdf]

miR-inhibitor	p-value	RZ-score(lfc)	lfc	MCF7	Resistant ($\bar{X}$)
**miR-423-3p**	1.445E-09	-5.94	-2.17	0.36	0.04
**miR-450b-3p**	1.361E-05	-4.20	-1.38	0.20	0.05
**miR-302b-3p**	1.099E-03	-3.06	-0.86	0.10	0.04
**miR-185-3p**	4.718E-03	-2.59	-0.65	0.10	0.05

Table S1: MiRs identified as modulating apoptosis in hormone-responsive (MCF7), but not tamoxifen-resistant (TamR) or aromatase inhibitor-resistant (long-term estrogen deprived [LTED]) breast cancer cells in high-throughput miR inhibitor screen. Lfc = log fold-change.

miR-inhibitor	p-value	RZ-score(lfc)	lfc	MCF7	Resistant ($\bar{X}$)
**miR-877-3p**	0.004098	2.64	1.72	0.11	0.61
**miR-593-5p**	0.002104	2.86	1.82	0.07	0.45
**miR-614**	0.005648	2.53	1.67	0.07	0.38
**miR-449b-3p**	0.000437	3.33	2.03	0.05	0.37
**miR-34a-3p**	0.000003	4.50	2.56	0.02	0.31
**miR-761**	0.001888	2.90	1.83	0.05	0.31
**miR-548x-3p**	0.001238	3.03	1.89	0.04	0.28
**miR-3123**	0.004336	2.62	1.71	0.05	0.27
**miR-3138**	0.002161	2.85	1.81	0.04	0.23

Table S2: MiRs identified as modulating apoptosis in Tamoxifen-resistant (TamR) or aromatase inhibitor-resistant (long-term estrogen deprived [LTED]), but not hormone-responsive (MCF7) breast cancer cells in high-throughput miR inhibitor screen. Lfc = log fold-change.

miR-inhibitor	p-value	RZ-score(lfc)	lfc	MCF7	Resistant ($\bar{X}$)
**miR-1228-3p**	0.1236	-1.16	-0.002	0.94	0.94
**miR-1260a**	0.1183	-1.18	-0.014	0.94	0.93
**miR-361-3p**	0.2137	-0.79	0.162	0.78	0.92
**miR-197-3p**	0.0965	-1.30	-0.068	0.89	0.83
**miR-885-5p**	0.0661	-1.51	-0.160	0.85	0.73
**let-7b-3p**	0.3334	-0.43	0.327	0.60	0.84
**miR-346**	0.1448	-1.06	0.042	0.73	0.76
**miR-1285-3p**	0.1018	-1.27	-0.054	0.72	0.68
**miR-1296-5p**	0.2777	-0.59	0.255	0.57	0.73
**miR-766-3p**	0.3665	-0.34	0.367	0.52	0.75
**miR-636**	0.1696	-0.96	0.089	0.62	0.68
**miR-1249-3p**	0.1718	-0.95	0.093	0.60	0.65
**miR-1273c**	0.0625	-1.53	-0.173	0.60	0.51

Table S3: MiRs identified as modulating apoptosis in Tamoxifen-resistant (TamR), aromatase inhibitor-resistant (long-term estrogen deprived [LTED]) and hormone-responsive (MCF7) breast cancer cells in high-throughput miR inhibitor screen. Lfc = log fold-change.

miR	MCF7	TamR	LTED
miR-423-3p	0.36	0.05	0.03
miR-614	0.07	0.66	0.10
miR-877-3p	0.11	0.81	0.41
miR-593-5p	0.07	0.73	0.18
miR-346	0.73	0.96	0.57
miR-361-3p	0.78	0.99	0.85

Table S4: MiRs selected from high-throughput miR inhibitor screen for further validation. Lfc = log fold-change.

Gene Symbol	LogFC	p-value	FDR
**ZBTB16**	3.58768449	0.00012443	0.01970354
**AZGP1**	2.93738319	3.70E-12	2.04E-08
**ADRA2A**	2.79170123	3.58E-05	0.00866378
**SLC26A2**	2.06093681	1.88E-06	0.00141849
**ELF5**	1.84946425	3.46E-05	0.00866378
**UGT2B11**	1.71693458	1.88E-07	0.00031142
**CNTD2**	1.5527896	8.79E-06	0.00373888
**MME**	1.51431272	0.00010845	0.01795773
**ACP5**	1.4501629	2.56E-09	7.05E-06
**RNU4-1**	1.42076376	0.0004872	0.04384295
**RPPH1**	1.3963751	2.83E-06	0.00173741
**PIP**	1.37640819	0.00035868	0.03666081
**CPB1**	1.34379086	2.05E-05	0.00649108
**RNU1-28P**	1.29294027	0.00029845	0.03384774
**RNVU1-7**	1.22610719	3.40E-05	0.00866378
**RMRP**	1.21748675	0.0003065	0.03429023
**RNU1-9**	1.21454995	2.19E-05	0.00655385
**RNU1-3**	1.21226078	1.22E-05	0.00469073
**RNU1-1**	1.21147373	4.91E-05	0.01041751
**MIR3687**	1.20525358	0.00026965	0.03184555
**RN7SK**	1.20462441	0.00040688	0.04034166
**SORBS1**	1.16488562	0.0002547	0.03101014
**TMEM178A**	1.15161341	0.00022766	0.02943237
**UGT2B15**	1.1119052	3.24E-08	7.66E-05
**MGP**	1.08354419	0.00017516	0.02391152
**MAT1A**	1.07935671	0.00051167	0.04530609
**SNORD3A**	1.07106101	3.89E-06	0.0020105
**MIR3648-2**	1.05175509	3.37E-05	0.00866378
**CRAT**	1.02944652	0.00033161	0.03519752
**SERPINA5**	1.02320642	3.48E-06	0.00191962
**IGFBP5**	1.02092072	4.51E-13	3.74E-09
**ERP27**	1.01312696	0.00041131	0.04037928
**RNU4-2**	1.0099447	0.00035121	0.03657409
**ANXA3**	-1.0026897	0.00024343	0.03023046
**AMIGO2**	-1.0470888	2.09E-10	8.64E-07
**TTLL7**	-1.3336169	2.64E-06	0.00173741
**TRIM47**	-1.353491	0.0005565	0.04799235
**ANKRD30A**	-1.4772583	2.55E-13	3.74E-09
**TGFB2**	-1.521355	0.00039042	0.03894361

Table S5: Top Differentially-Expressed Genes Identified by RNA-seq Analysis of MCF7 Xenograft Tumours from Balb/c Mice Treated with 10mg/kg ASO-361-3p versus ASO-NC for 18d.

Gene Symbol	Log2-FC	p-value	FDR
**ACP5**	1.450162901	2.56E-09	7.05E-06
**NCAPH**	-0.66398803	1.65E-06	0.00137
**FAM72D**	-0.7484549	2.34E-06	0.00162
**ALDH3B2**	0.864166003	3.11E-06	0.00181
**FAM72A**	-0.63421833	5.08E-06	0.00252
**FAM72B**	-0.67646553	3.82E-05	0.00895
**FANCA**	-0.66704175	0.000113	0.01853
**POLQ**	-0.56576052	0.000119	0.01932
**ZBTB16**	3.587684494	0.000124	0.0197

Table S6: Differentially-Expressed Genes from ASO-361-3p- versus ASO-NC-Treated MCF7 Xenografts that were Identified as Potentially Direct MiR-361-3p Targets from MiR Target Databases and Breast Cancer AGO-PAR-CLIP-seq Datasets. Log2FC, p-value and FDR refers to log2 fold change, p-value and false discovery rate-adjusted p-value in RNA-seq dataset.

Sample	Tumour type	Age	Menopause Status	Side	Size (mm)	Grade	Tumour Type	ER	PR	HER2	LNs	Surgery Type
**Mi06**	Primary	59	Post	Left	90	2	ILC	8/8	8/8	Neg	0/1	MX
**Mi07**	Primary	34	Pre	Right	28	2	IDC + HGDCIS	8/8	8/8	Neg	0/3	WLE
**Mi08**	Primary	45	Pre	Right	31	2	Multi-focal IDC + IG and HG DCIS	8/8	8/8	Neg	0/3	Mx

Table S7: Clinical and Pathological Characteristics of Breast Cancer Patients from whom Tissues were Obtained for Explants. LN: lymph node, ILC: invasive lobular carcinoma, IDC: invasive ductal carcinoma, IG/HG DCIS: intermediate-grade/ high-grade ductal carcinoma *in situ*, Mx: mastectomy, WLE: wide local excision, ER: estrogen receptor. ER evaluation value corresponds to Alfred score attributed by immunohistochemistry, which combines both the percentage of positive cells for the receptor, as well as staining intensity. Score 0-2 are considered negative, and 3-8 positive; PR: progesterone receptor. The PR evaluation values are attributed as for ER.
